# Beyond the Lindlar catalyst: highly-oxidized Pd single atoms as promoters for alkyne semi-hydrogenation

**DOI:** 10.1039/d5sc08632a

**Published:** 2026-01-06

**Authors:** Ming Jiang, Yao Lv, Zhongzhe Wei, Xu Liu, Zhixiang Yang, Chuanming Chen, Yiming Hu, Fangjun Shao, Xiaonian Li, Jiaxing Hu, Sheng Dai, Jianguo Wang

**Affiliations:** a State Key Laboratory of Green Chemical Synthesis and Conversion, Zhejiang Key Laboratory of Surface and Interface Science and Engineering for Catalysts, College of Chemical Engineering, Zhejiang University of Technology Hangzhou 310032 China weizhzhe@zjut.edu.cn jgw@zjut.edu.cn; b Key Laboratory for Advanced Materials and Feringa Nobel Prize Scientist Joint Research Center, School of Chemistry and Molecular Engineering, East China University of Science & Technology Shanghai 200237 P. R. China shengdai@ecust.edu.cn; c Zhejiang Huahai Pharmaceutical Co. Ltd Linhai 317024 P. R. China hujiaxing@huahaipharm.com

## Abstract

The semi-hydrogenation of alkynes is crucial for the synthesis of steroid hormone drugs, yet conventional approaches relying on Pd surface poisoning additives sacrifice activity and sustainability for selectivity. Herein, we present a “grafting-then-coordination” strategy to construct a Pd/C–NH_2_ catalyst featuring coexisting tetravalent Pd single atoms (Pd_IV_ SAs) and Pd nanoparticles (NPs), which achieves inhibitor-free and highly efficient hydrogenation of steroidal alkynes. The Pd/C–NH_2_ catalyst, functionalized with 3-aminopropyltriethoxysilane (APTES), exhibits 99% conversion with 97% selectivity in the selective hydrogenation of mifepristone under 0.1 MPa at 25 °C, with a remarkable turnover frequency (TOF) of 3675 h^−1^, representing a 17-fold enhancement over the conventional Lindlar catalyst. Mechanistic studies reveal that the Pd_IV_ SAs are stabilized through Pd–N/O coordination by leveraging oxygen-containing groups of the support and amino groups of the ligand. The electron-deficient Pd_IV_ SAs adsorb mifepristone, mitigating substrate self-poisoning on Pd NPs, while Pd NPs activate H_2_ and promote hydrogen spillover to Pd_IV_ SAs, enabling hydrogenation *via* a dual-site cooperative mechanism. The stable Pd_IV_ SAs transform conventional poisoning sites into productive active centers, offering valuable insights for the rational design of advanced selective hydrogenation catalysts.

## Abstract 2

A “grafting-then-coordination” strategy was proposed to construct Pd/C-NH_2_ featuring coexisting tetravalent Pd single atoms (Pd_IV_ SAs) and Pd nanoparticles (Pd NPs), which achieves inhibitor-free, highly efficient hydrogenation of steroidal alkynes.

## Graphical abstract

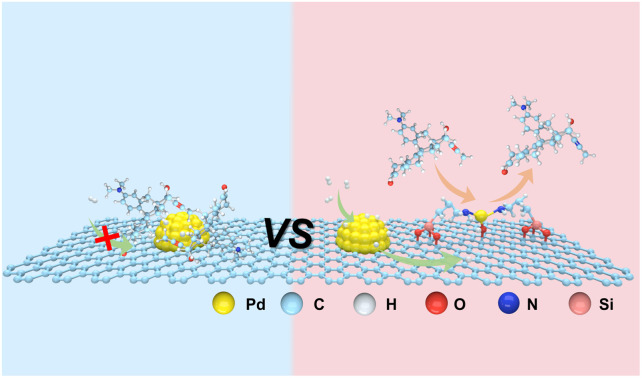

## Introduction

The semi-hydrogenation of alkynes is crucial for the synthesis of fine chemicals, pharmaceuticals, and petrochemicals^1–3^ and is particularly indispensable in the production of steroid hormone drugs.^4^ In 2016, global sales of steroid hormone medications surpassed $100 billion, making them the second-largest class of drugs after antibiotics,^5^ reflecting their critical role in regulating biological functions. Aglepristone, the semi-hydrogenated derivative of mifepristone, acts as a progesterone antagonist^6^ and is widely employed in the treatment of insulin-resistant diabetes and acromegaly. Product selectivity directly influences the final purity, yield, and economic feasibility of the manufacturing process.^7,8^ Compared to simpler alkynes such as phenylacetylene, steroidal alkynes possess a polycyclic structure that leads to stronger adsorption on catalyst surfaces, exacerbating the competitive adsorption–desorption dynamics between alkynes and alkenes.^9–11^ Furthermore, the steric bulk of mifepristone-derived molecules introduces mass transfer constraints that hinder access to active sites.^12–14^ Consequently, the hydrogenation process faces dual challenges of low selectivity and insufficient activity. Thus, the development of efficient catalysts for the semi-hydrogenation of steroidal alkynes, along with a deeper understanding of how the catalysts modulate adsorption and desorption behavior, remains a central goal in this field.

Pd-based catalysts are widely used in alkyne semi-hydrogenation due to their excellent ability in H_2_ activation. However, the strong adsorption of alkynes on continuous Pd surfaces often leads to over-hydrogenation.^11,15,16^ In industrial catalysis, the Lindlar catalyst^17–19^ is regarded as a benchmark for alkyne semi-hydrogenation. Although it offers good selectivity, its Pb-containing components pose environmental risks and significantly reduce both catalytic activity and metal utilization, limiting its industrial applicability. Alternatively, strategies such as alloying to modify electronic properties,^15,20–22^ doping with inexpensive metals,^23–25^ or partial poisoning of active centers^26–29^ have been employed to disrupt the Pd ensemble effect and thereby promote alkene desorption. Unfortunately, these approaches usually result in reduced activity and lower metal utilization^30,31^ Recent studies show that single-atom (SA) catalysts are highly effective in promoting alkene desorption.^32–34^ Their isolated sites not only eliminate the geometric effects associated with multi-site adsorption^35^ but also feature electron-deficient metal centers^36^ that reduce electronic back-donation to olefin products. This weakens the adsorption strength of alkenes and facilitates their desorption, thereby enhancing selectivity towards alkenes. Studies have shown that the valence state of Pd SAs can be tuned between 0 and +2;^33,34,37,38^ however, the specific catalytic mechanism of more electron-deficient states (such as Pd^4+^) in hydrogenation reactions remains unclear. More critically, unstable high-valent Pd^4+^ species have only been observed in certain complexation scenarios, typically with the combination of potential oxidants^39,40^ such as mer-tridentate carbene pincers and chelating bipyridyl. Furthermore, under reductive reaction atmospheres, Pd^4+^ is prone to reduction or aggregation, leading to structural degradation.^40^ Therefore, the controllable synthesis and long-term stability of Pd_IV_ SAs still face significant challenges. Moreover, Pd SAs with isolated sites exhibit poor H_2_ dissociation capability^41,42^ and often require harsh reaction conditions to achieve efficient hydrogenation. In the hydrogenation of mifepristone, its polycyclic structure tends to strongly adsorb on Pd surfaces, blocking active sites and making efficient H_2_ activation particularly crucial. Constructing a catalytic system with coexisting single atoms and nanoparticles may synergistically enhance both activity and selectivity, offering a promising path to overcome current limitations.

This work presents a ligand modification strategy for the controlled construction of a synergistic catalytic system with coexisting Pd_IV_ SAs and Pd NPs, achieving highly efficient and selective hydrogenation of mifepristone. Leveraging the abundant surface hydroxyl (–OH) and carbonyl (C=O) groups on the carbon support, a precise “grafting-then-coordination” mechanism is established: under aqueous conditions, surface hydroxyl groups facilitate the hydrolysis and condensation of 3-aminopropyltriethoxysilane (APTES), forming covalent Si–O–C bonds that graft amino groups (–NH_2_) onto the carrier. Subsequently, C=O and –NH_2_ groups act as coordination sites to stabilize Pd species, forming structurally stable Pd_IV_ SAs. This approach allows quantitative tuning of the active site ratio, thereby optimizing the synergistic interplay between Pd_IV_ SAs and Pd NPs. Under mild conditions (25 °C, 0.1 MPa), the optimized Pd/C–NH_2_ catalyst exhibits exceptional performance in the semi-hydrogenation of mifepristone, achieving 99% conversion, 97% selectivity, and a TOF of 3675 h^−1^, significantly outperforming the performance of commercial Pd/C and the Lindlar catalyst. Mechanistic investigations reveal that Pd_IV_ SAs adsorb mifepristone, mitigating substrate self-poisoning on Pd NPs, while Pd NPs activate H_2_ and promote hydrogen spillover to hydrogen-deficient Pd_IV_ SAs, collectively enabling hydrogenation *via* a dual-site cooperative mechanism. The ligand modification strategy not only stabilizes metastable Pd_IV_ SAs but also alleviates competitive adsorption between H_2_ and mifepristone, ultimately enabling highly efficient and selective hydrogenation.

## Experimental

### Synthesis of C–NH_2_-*t*

500 mg of activated carbon was dispersed in 50 mL of deionized water and subjected to ultrasonication for 30 min. Then, 3-aminopropyltriethoxysilane (APTES) was added, and the mixture was stirred at 35 °C for 12 h, with a mass ratio of carrier to modifier fixed at 1 : 2. The resulting product was filtered under vacuum, thoroughly washed with deionized water until the filtrate reached neutral pH and dried overnight at 70 °C to obtain C–NH_2_. Using the same conditions and an equivalent amount of modifier, a series of APTES-modified carriers designated as C–NH_2_-*t* (where *t* denotes the stirring time with the modifier: *t* = 5, 12, and 24 h) were prepared under different modification durations. Furthermore, the mass ratio between activated carbon and APTES, as well as the type of silane coupling agent, was systematically screened.

### Synthesis of Pd/C–NH_2_-*t*

A series of Pd-based catalysts were prepared using the impregnation method. Specifically, 500 mg of C–NH_2_-*t* support was dispersed in 30 mL of deionized water and ultrasonicated for 30 min. An aqueous solution of K_2_PdCl_4_ (containing 20 mg per mL Pd) was added, and the mixture was stirred at 35 °C for 1 h. Then, 10 mL of a freshly prepared sodium borohydride solution (20 mg mL^−1^) was added, with stirring continued for 1 h. The resulting solid was collected by vacuum filtration, washed thoroughly, and dried at 70 °C for 12 h to obtain Pd/C–NH_2_-*t*. For comparison, a reference catalyst Pd/C was synthesized using the same procedure with unmodified activated carbon as the support. Additional control catalysts were also prepared *via* the same impregnation approach using supports synthesized under different conditions. The theoretical Pd loading in all catalysts was 5 wt%.

## Results and discussion

### Fabrication and characterization of catalysts

**Fig. 1:**
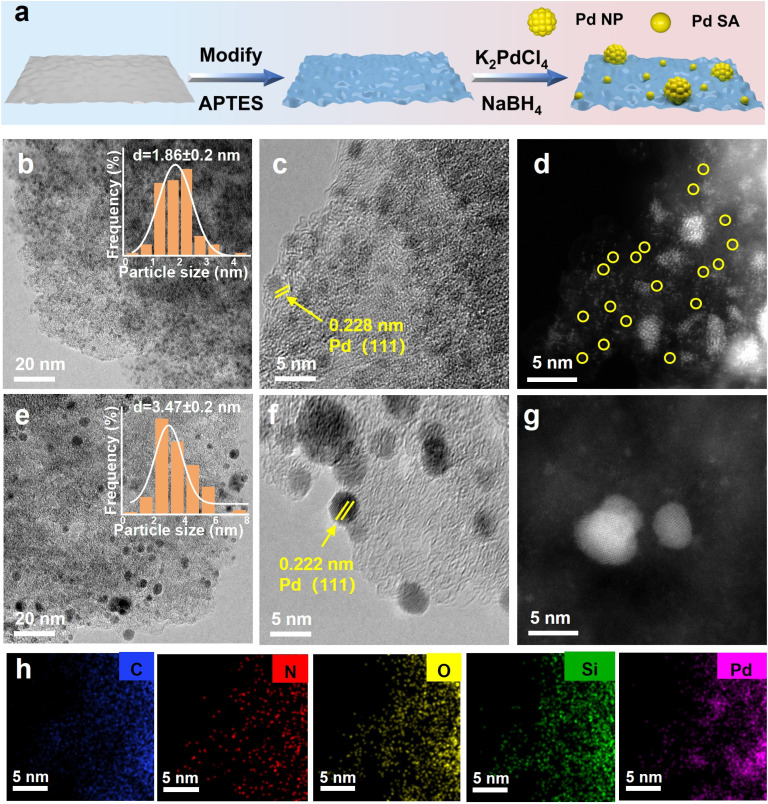
Characterization of Pd/C–NH_2_ and Pd/C. (a) Schematic illustration for the synthetic processes of Pd/C–NH_2_. (b, c, e and f) TEM and HRTEM images of Pd/C–NH_2_ and Pd/C. (d and g) AC-STEM images of Pd/C–NH_2_ and Pd/C. (h) EDS elemental distribution diagram of Pd/C–NH_2_.

The simultaneous and controlled distribution of Pd_IV_ SA and Pd NP active centers on activated carbon was achieved through a stepwise process involving initial grafting of functional ligands onto the support, followed by precise metal coordination and reduction, as illustrated in Fig. 1a. The carbon support rich in oxygen-containing functional groups was first modified with APTES. The surface hydroxyl groups on the carbon guide the hydrolysis of APTES, leading to the elimination of an ethanol molecule and formation of covalent Si–O–C bonds, thereby grafting –NH_2_ groups onto the carbon framework.^43^ The introduction of –NH_2_ plays a crucial role in controlling the dispersion of metal nanostructures,^44^ which is beneficial for the formation of uniformly distributed and smaller-sized Pd species. Subsequent wet impregnation with an aqueous K_2_PdCl_4_ solution and reduction with NaBH_4_ yielded a series of catalysts labeled as Pd/C–NH_2_-*t* (where *t* represents the APTES modification time). The optimal catalyst, Pd/C–NH_2_, was obtained after 12 h of APTES modification.

Inductively coupled plasma optical emission spectrometry (ICP-OES) analysis confirmed Pd loadings of 4.95% for Pd/C and 4.93% for Pd/C–NH_2_ (Table S1), indicating no substantial metal loss during functionalization. High-resolution transmission electron microscopy (HRTEM) and high-angle annular dark-field scanning transmission electron microscopy (HAADF-STEM) images, combined with aberration-corrected transmission electron microscopy (AC-STEM) results (Fig. 1b–g, and S1–6), revealed a broad size distribution of Pd NPs in the unmodified Pd/C catalyst, with an average size of 3.47 nm. The observed lattice spacing of 0.222 nm corresponds to the Pd (111) plane. In contrast, the APTES-modified Pd/C–NH_2_-*t* catalyst exhibits a higher density of finely dispersed and narrowly distributed Pd NPs, along with evidence of Pd SAs. Notably, Pd/C–NH_2_ shows Pd NPs averaging only 1.86 nm in size, with an observed interplanar spacing of 0.228 nm corresponding to the Pd (111) surface. These results indicate that the –NH_2_ ligand modification significantly enhances the dispersion of Pd. Electron microscopy observations with aberration corrected transmission electron microscopy (Fig. 1d and g) demonstrate that the APTES-modified catalyst not only carries smaller Pd NPs but also contains abundant Pd SAs. Energy-dispersive X-ray (EDX) analysis in Fig. 1h reveals a homogeneous distribution of C, N, O, Si and Pd elements throughout the catalyst. The intensified nitrogen signal confirms the successful incorporation of –NH_2_ groups into the support. Hydrogen–oxygen titration measurements (H_2_–O_2_) indicated a remarkable increase in Pd dispersion from 15.55% to 43.31% after APTES modification (Table S2), unequivocally demonstrating that APTES functionalization substantially improves the dispersion of Pd species on the carbon support. To determine the valence states of the Pd atoms, spherical aberration-corrected transmission electron microscopy combined with electron energy loss spectroscopy (EELS) was employed to characterize the Pd/C–NH_2_ composite (Fig. S7). As EELS analysis at the single-atom level was not feasible, the investigation focused specifically on Pd NPs. The observed high-energy shift in the EELS spectra indicated the presence of Pd atoms in higher oxidation states.^45^ Three distinct Pd NP models exhibited EELS profiles with energy positions situated between those of the Pd^0^ and Pd^2+^ reference standards, suggesting that the bonded Pd atoms in Pd NPs are in an oxidation state of Pd^*δ*+^ (0 < *δ* < +2).

**Fig. 2:**
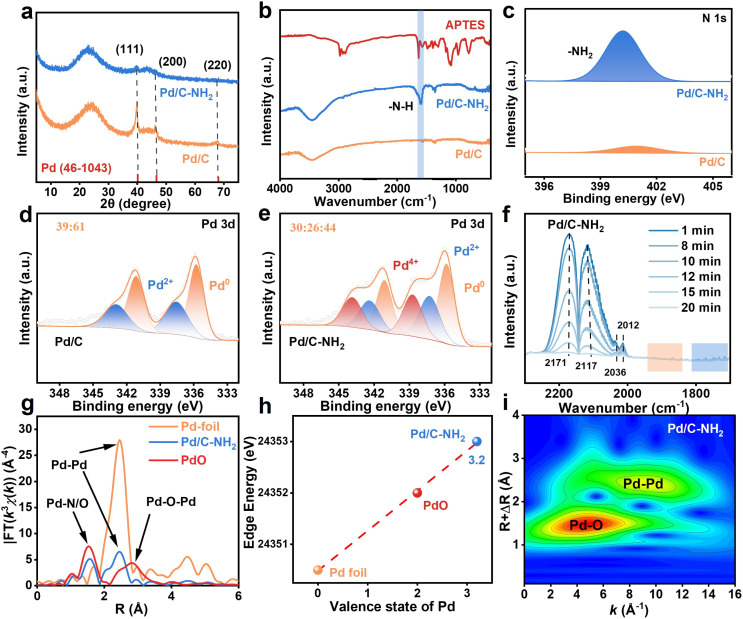
Structure characterization of Pd/C–NH_2_. (a) XRD patterns of Pd/C and Pd/C–NH_2_. (b) FTIR spectra of APTES, Pd/C and Pd/C–NH_2_. (c–e) High-resolution N 1s and Pd 3d XPS spectra of Pd/C and Pd/C–NH_2_. (f) CO-DRIFTS spectra of Pd/C–NH_2_. (g) FT-EXAFS spectra of Pd/C–NH_2_, Pd foil, and PdO. (h) The average oxidation states of Pd species in the catalysts determined by Pd K-edge XANES analysis. (i) Wavelet-transformed spectra of Pd/C–NH_2_.

X-ray diffraction (XRD) patterns (Fig. 2a, and S8) of Pd/C and Pd/C–NH_2_ exhibit a characteristic peak at near 2*θ* = 23°, corresponding to the (002) plane of the carbon material, indicating a high degree of graphitization.^46^ The distinctive peaks observed at 2*θ* = 40°, 46°, and 68° for Pd/C are assigned to the (111), (200), and (220) planes of metallic Pd,^47^ respectively. As the time for APTES modification increased, the intensity of the Pd peaks gradually diminished and eventually disappeared, indicating a reduction in particle size. This phenomenon is consistent with the results of electron microscopy. Raman spectroscopy (Fig. S9 and Table S3) further analyzed the carbon structure of the two catalysts. Signals at 1345–1351 cm^−1^ and 1597–1603 cm^−1^ correspond to the D-band (defect-related) and G-band (graphitization-related), respectively. The nearly identical *I*_D_/*I*_G_ ratio of Pd/C–NH_2_ (2.27) and Pd/C (2.25) indicated that the APTES modification did not affect the graphitization level of the carbon framework.^48^ Fourier transform infrared spectroscopy (FT-IR) confirmed the successful grafting of APTE, as evidenced by a new absorption band at 1597 cm^−1^ in Pd/C–NH_2_-*t*, attributed to the –N–H stretching vibration of the amino-silane (Fig. 2b and S10).^43^ N_2_ adsorption–desorption isotherms shown in Fig. S11 and Table S4 revealed a reduction in specific surface area and pore volume after APTES modification, but an increased mean pore size (from 3.8 to 6.9 nm), which can promote the diffusion of mifepristone considering its large steric hindrance. After Pd loading, the catalysts showed similar specific surface areas and pore sizes with the support, preserving a mesoporous structure. X-ray photoelectron spectroscopy (XPS) was employed to explore the surface composition and electronic interactions between components of catalysts (Fig. 2c–e, and S12–16). The appearance of N 1s and Si 2p signals after modification further confirmed the successful introduction of APTES. High-resolution O 1s spectra distinguishes 529.6 eV for lattice oxygen (O_latt_) from 532.1 eV for adsorbed oxygen (O_ads_).^28^ The high-resolution C 1s spectrum exhibits distinct peaks at 286.3 eV and 288.2 eV, corresponding to –C–O and C=O bonds, respectively. The abundance of oxygen-containing functional groups in Pd/C–NH_2_ facilitates APTES grafting and subsequently coordination with Pd to form Pd_IV_ SAs. High-resolution Pd 3d spectra confirmed the coexistence of Pd^0^ and Pd^2+^ species in Pd/C, with characteristic binding energies observed at 335.3 eV (Pd^0^ 3d_5/2_), 340.6 eV (Pd^0^ 3d_3/2_), 336.5 eV (Pd^2+^ 3d_5/2_), and 342.8 eV (Pd^2+^ 3d_3/2_). Notably, two additional peaks appeared at 338.3 eV and 344.5 eV in Pd/C–NH_2_, exhibiting intensities even higher than those of the Pd^2+^ species. These peaks can be assigned to the 3d_5/2_ and 3d_3/2_ orbitals of Pd^4+^, respectively.^49,50^ In the unmodified Pd/C catalyst, Pd^0^ constitutes 61% of the Pd species, whereas in Pd/C–NH_2_, the proportion of Pd^0^ decreases to 44%, with Pd^4+^ accounting for 30%. The presence of Pd^2+^ is commonly attributed to the surface oxidation during synthesis. Interestingly, the APTES-modified catalyst exhibited an even higher oxidation state of Pd^4+^ than Pd^2+^, and the proportion of Pd^4+^ increased as the APTES modification time extended. EELS analysis showed that Pd NPs primarily exhibit oxidation states between 0 and +2, suggesting that the observed Pd^4+^ species in Pd/C–NH_2_ originate from atomically dispersed Pd, indicative of the successful formation of Pd_IV_ SAs after modification. Unexpectedly, XPS analysis of the catalyst Pd/C–NH_2_-unreduced (synthesized identically but without NaBH_4_ reduction) also revealed that it contained 34% Pd^4+^ (Fig. S17). This suggests that Pd^4+^ species form during the loading step and remain stable even under the strong reducing conditions of NaBH_4_ treatment. The local environment of palladium species on the support was further characterized by CO-DRIFT spectroscopy (Fig. 2f, and S18). The characteristic peaks observed at 2170 cm^−1^ and 2117 cm^−1^ correspond to free CO molecules. Notably, the peaks at 2036 cm^−1^ and 2012 cm^−1^ for Pd/C–NH_2_ showed significantly higher intensity than those for Pd/C. The peak at 2036 cm^−1^ can be assigned to the symmetric stretching vibration of gem-dicarbonyl doublet CO on positively charged Pd species, while the peak at 2012 cm^−1^ corresponds to the stretching vibrations of linearly adsorbed CO on isolated Pd sites and bridge-adsorbed CO on ultrasmall Pd clusters. Additionally, the characteristic peaks in the 1840–1940 cm^−1^ range indicate the presence of bridge-adsorbed CO on Pd nanoparticles, while those in the 1700–1820 cm^−1^ range are attributed to triply coordinated adsorbed CO. These spectral features confirm that the modification treatment successfully constructed isolated Pd SAs in addition to the original Pd NPs. These spectroscopic assignments align well with AC-STEM imaging data.^51,52^

To gain deeper insight into the local coordination environment of Pd, X-ray absorption fine structure (XAFS) measurements were conducted at the Pd K-edge.^49,50^ As shown in Fig. 2g, Fourier transform (FT) *K*^2^-weighted EXAFS analysis of Pd/C–NH_2_ shows a pronounced coordination peak at approximately 2.74 Å in *R*-space, corresponding to Pd–Pd scattering paths. This peak is markedly weaker than that of the electron transfer. The *k*-space EXAFS spectra and their corresponding fits for Pd/C–NH_2_ and reference samples display similar oscillatory behavior in the low-*k* region (3–9 Å^−1^). The high accuracy of the fitting was validated by an *R*-factor of 0.0075, significantly lower than the reference threshold of 0.024748. Wavelet transform (WT) EXAFS analysis (Fig. 2i) further discriminates these bonding types. The wavelet contour plot of Pd/C–NH_2_ exhibits maximum intensity at 2.0–3.0 Å and 9.0 Å^−1^, indicative of Pd–Pd coordination, alongside a new feature at 1.0–2.0 Å and 4.2 Å^−1^, corresponding to Pd–N/O coordination. This confirms that Pd species are coordinated not only to each other but also to N/O atoms from the support. Quantitative EXAFS fitting (Fig. S19 and Table S5) provided coordination numbers of 3.1 ± 0.2 for Pd–N/O and 3.0 ± 0.1 for Pd–Pd, with bond lengths of 2.028 ± 0.005 Å and 2.739 ± 0.004 Å, respectively. XAFS data and fitting results demonstrates that Pd/C–NH_2_ contains both Pd–N/O and Pd–Pd coordination environments, indicating the presence of both Pd SAs and small-sized Pd NPs in Pd/C–NH_2_. Furthermore, by establishing a linear relationship between absorption edge energy and known valence states of Pd foil and PdO, the average oxidation state of Pd in Pd/C–NH_2_ was effectively semi-quantitatively determined to be +3.2 (Fig. 2h). Given that EELS analysis indicated that the valence state of Pd NPs is between 0 and +2, an average oxidation state of +3.2 significantly exceeds the expected range for metallic Pd or PdO nanoparticle-based configurations. This suggests the presence of Pd species with a valence state higher than +3.2 in the system. Combined with the observation of 30% Pd^4+^ content in XPS characterization, it further confirms that the Pd SAs in the catalyst are in the +4 oxidation state. Therefore, ligand modification enables the synthesis of a catalyst containing stable Pd_IV_ SAs, as unequivocally confirmed by complementary XAFS, XPS, and EELS analyses.

### Study on the formation mechanism of Pd_IV_ SAs

**Fig. 3:**
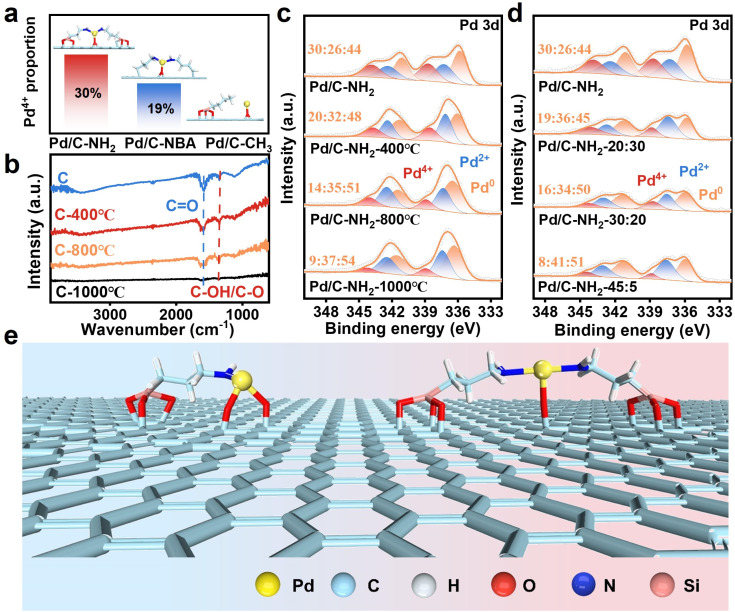
Formation mechanism of Pd^4+^ in Pd/C–NH_2_. (a) The proportion of Pd^4+^ in catalysts modified with different silane coupling agents. (b) FT-IR spectra of catalysts with supports calculated at different temperatures. (c) High-resolution Pd 3d XPS spectra of catalysts with supports calculated at different temperatures. (d) High-resolution Pd 3d XPS spectra of catalysts with different *V*_EtOH_ : *V*_H_2_O_ ratios for hydrolysis. (e) Schematic diagram of the grafting form of the ligand, support and Pd in Pd/C–NH_2_.

Pd^4+^ is notoriously difficult to form under conventional conditions, and it typically exists as a highly unstable intermediate^39,40,53^ when it does occur. Remarkably, in our system, we have successfully synthesized stable Pd_IV_ SAs, making it essential to elucidate their formation mechanism. Preliminary experiments indicated that the introduction of APTES is crucial for the formation of Pd_IV_ SAs. APTES, a silane coupling agent, contains both an –NH_2_ terminal and a hydrolyzable –Si–O terminal.^43^ To clarify the role of these functional groups, we modified the support using *n*-butylamine (which possesses only an –NH_2_ group) to prepare the Pd/C–NBA catalyst. XPS characterization revealed that although Pd_IV_ formed in Pd/C–NBA, the proportion of Pd^4+^ was 19%, significantly lower than that in Pd/C–NH_2_. Nevertheless, this confirms that Pd^4+^ can still form under these conditions. Subsequently, we used *n*-propyltriethoxysilane, which lacks an –NH_2_ group, to synthesize the Pd/C–CH_3_ catalyst. Interestingly, no Pd^4+^ was detected in Pd/C–CH_3_, indicating that the –NH_2_ group is indeed indispensable for the formation of Pd^4+^ (Fig. 3a, S20 and 21). Furthermore, the effect of the –NH_2_ group is closely related to the hydrolysis degree of the silane grafted on the carbon support. Therefore, we proposed to control the content of Pd_IV_ SAs by regulating the hydrolysis degree of the silane coupling agent. This was approached from two perspectives: first, the oxygen-containing functional groups on the carbon surface were reduced by high-temperature calcination under an inert atmosphere. We observed that the Pd^4+^ content decreased significantly with increasing calcination temperature (Fig. 3c). In the FT-IR spectra (Fig. 3b), the characteristic peaks at 1345 cm^−1^ and 1590 cm^−1^ are attributed to the stretching vibrations of C–OH/C–O and C=O bonds,^54^ respectively. The intensity of these peaks gradually weakened with increasing calcination temperature and almost vanished at 1000 °C, indicating extensive removal of oxygen-containing functional groups. After the carbon was calcined at 1000 °C and treated with APTES under identical modification conditions, only 9% of Pd^4+^ was detected in Pd/C–NH_2_-1000 °C, demonstrating that oxygen-containing functional groups on the support surface are critical for APTES hydrolysis. That is, the carbon support possesses sufficient –OH and C=O groups to facilitate both APTES hydrolysis/grafting and subsequent Pd–N/O coordination. Additionally, the hydrolysis rate was adjusted by varying the ratio of ethanol to water during hydrolysis. As expected, the introduction of ethanol may inhibit the hydrolysis of APTES.^55^ When the modification was carried out with an ethanol-to-water volume ratio of 45 : 5, only 8% of Pd^4+^ species were formed (Fig. 3d). This indicates that the hydrolysis–condensation reaction of APTES proceeds most rapidly under pure aqueous conditions, resulting in the highest grafting density of –NH_2_ groups on the carbon support and consequently leading to the highest content of Pd^4+^.

Based on the above findings, we conclude that the formation and stabilization of Pd_IV_ SAs arise from the synergistic interplay between oxygen-containing functional groups on the support surface and the –NH_2_ groups from the ligand. EXAFS fitting revealed a Pd–N/O coordination number of 3.1, indicating that Pd_IV_ SAs are stabilized through coordination with both N from the ligand and O from the carrier.^29^ We therefore propose that Pd_IV_ SAs are coordinated in either a [Pd–N_1_O_2_] or [Pd–N_2_O_1_] configuration (Fig. 3e). The “grafting-then-coordination” regulation mechanism enables precise Pd–N/O coordination. Under aqueous conditions, the –OH groups on the carrier surface promote the hydrolysis and condensation of APTES, forming covalent Si–O–C bonds and thereby grafting –NH_2_ onto the carbon support. Subsequently, C=O and –NH_2_ groups act as coordination sites to anchor Pd species into a well-defined geometry, leading to the formation of stable Pd_IV_ SAs. By controlling the concentration of oxygen species on the support as well as the hydrolysis rate of the silane ligand, the proportion of SA active sites can be quantitatively tailored, enabling precise regulation of the synergistic catalysis between Pd_IV_ SAs and Pd NPs.

### Catalytic performance

**Fig. 4:**
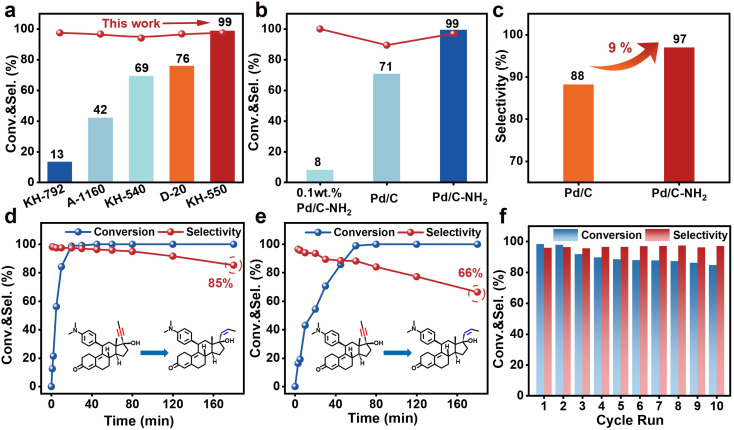
Catalytic activity evaluation. (a) Comparison of catalytic performance of catalysts modified with different silane coupling agents. (b) Comparison of conversion of Pd/C–NH_2_, Pd/C and 0.1 wt% Pd/C–NH_2_. (c) Comparison of selectivity between Pd/C–NH_2_ and Pd/C. (d and e) Time curves of Pd/C–NH_2_ and Pd/C. (f) Stability cyclic test of Pd/C–NH_2_. Reaction conditions: 250 mg mifepristone, 6 mg catalyst, 25 °C, 0.1 MPa.

This study evaluated the performance of the synthesized catalysts in the semi-hydrogenation of mifepristone. To eliminate the influence of mass transfer on the reaction rate, the semi-hydrogenation of mifepristone was conducted under varying catalyst mass (internal diffusion) and stirring speed (external diffusion). As shown in Fig. S22 and S23, the conversion of mifepristone showed a linear correlation with catalyst mass. When the stirring speed exceeded 800 rpm, the conversion rate remains essentially unchanged, indicating that mass transfer limitations are negligible under the current reaction conditions. As illustrated in Fig. 4a, a comparison among different silane coupling agents revealed that APTES exhibited superior activity and selectivity under the same treatment conditions. By optimizing the ligand content and modification time (Fig. S24 and S25), it was found that the optimal mass ratio of carbon to APTES was 1 : 2, beyond which further increases in ligand content had minimal impact on catalytic performance. Catalytic activity increased with longer modification time, while the selectivity remained consistently above 93%, which further verifies the promoting effect of APTES modification on the catalytic performance. Consequently, the optimal catalyst preparation conditions were determined as follows: *m*_C_ : *m*_APTES_ = 1 : 2, with APTES modification conducted at 35 °C for 12 h. Next, we focused on comparing the catalytic performance of the Pd/C and Pd/C–NH_2_ catalysts. As shown in Fig. 4b, c and S26, under identical reaction conditions (25 °C, 0.1 MPa), the Pd/C–NH_2_ catalyst achieved 99% conversion–a 28% improvement compared to the 71% conversion obtained with the unmodified Pd/C catalyst. Furthermore, the selectivity increased from 88% (Pd/C, containing 7% *trans*-olefins and 5% over-hydrogenated byproducts) to 97% (Pd/C–NH_2_, containing 2% *trans*-olefins and 1% over-hydrogenated byproducts) at full conversion. At a low conversion level (conversion < 30%, Fig. S27, and Table S6), the TOF of Pd/C–NH_2_ reaches 3675 h^−1^, which is 17-fold higher than that of the Lindlar catalyst (205 h^−1^) and outperforms commercial Pd/C (2695 h^−1^) and Pd/C (3094 h^−1^). In comparison, the 0.1 wt% Pd/C–NH_2_ catalyst achieved a mere 8% conversion for mifepristone, with a TOF of only 18 h^−1^. Moreover, during the hydrogenation of phenylacetylene, Pd/C–NH_2_ also demonstrates a performance advantage over some of the currently reported catalysts for phenylacetylene semi-hydrogenation (Table S7). AC-STEM imaging showed that the 0.1 wt% Pd/C–NH_2_ catalyst contained mainly small Pd NPs along with a significant number of Pd SAs (Fig. S28 and S29). XPS analysis further revealed that 59% of the Pd species in the catalyst were present as Pd^4+^ (Fig. S30). This indicated that the sole Pd SAs exhibit intrinsic limitations in the hydrogenation reaction of mifepristone.

The time–course profile of Pd/C–NH_2_ (Fig. 4d) showed that complete conversion of mifepristone was achieved within 30 min with 97% selectivity. In contrast, Pd/C required 60 min to reach full conversion with only 88% selectivity (Fig. 4e), demonstrating its inferior performance. When the reaction was extended to 180 min, Pd/C–NH_2_ still retained 85% selectivity, whereas that of Pd/C decreased significantly to 66%, underscoring the good selectivity for Pd/C–NH_2_. Therefore, the simultaneous enhancement in both activity and selectivity was achieved through the APTES-modified support, enabled by the coexistence of Pd_IV_ SA and Pd NP active sites. Beyond catalytic activity and selectivity, the stability of Pd/C–NH_2_ and Pd/C was assessed through reuse over multiple reaction cycles (Fig. 4f, and S31). The Pd/C–NH_2_ catalyst exhibited relatively stable performance over 10 consecutive reaction cycles, with activity declining from 99% to 85% while selectivity remained unchanged. This decrease in activity is likely attributable to valence state instability under H_2_ reaction conditions. In contrast, Pd/C underwent more severe deactivation under identical cycling conditions, decreasing from 99% to 60%, which indicates that the stability of Pd/C–NH_2_ is significantly superior to that of Pd/C. TEM observations showed that the particle size of Pd/C–NH_2_ increased from 1.86 to 1.93 nm after recycling, with no significant aggregation detected, confirming the strong anchoring effect of –NH_2_ ligands (Fig. S32–37). In contrast, Pd/C exhibited remarkable aggregation of Pd particles (>10 nm), which is the main cause of Pd/C deactivation. After recycling, Pd/C–NH_2_ retained the characteristic infrared absorption peak of –NH_2_ (Fig. S38), indicating that the ligands were firmly anchored and did not leach during the reaction. ICP-OES data showed that the Pd contents for the reused Pd/C–NH_2_ and Pd/C are 4.71 wt% and 3.36 wt% (Table S1), respectively, which further demonstrating the strong anchoring effect of –NH_2_ groups. XPS analysis of the recycled catalysts revealed that Pd^4+^ in Pd/C–NH_2_ was partially reduced (Fig. S39), which would diminish the contribution of the Pd^4+^ mediated selective hydrogenation pathway and result in decreased catalytic activity.

### Investigation on the action mechanism of Pd_IV_ SAs

**Fig. 5:**
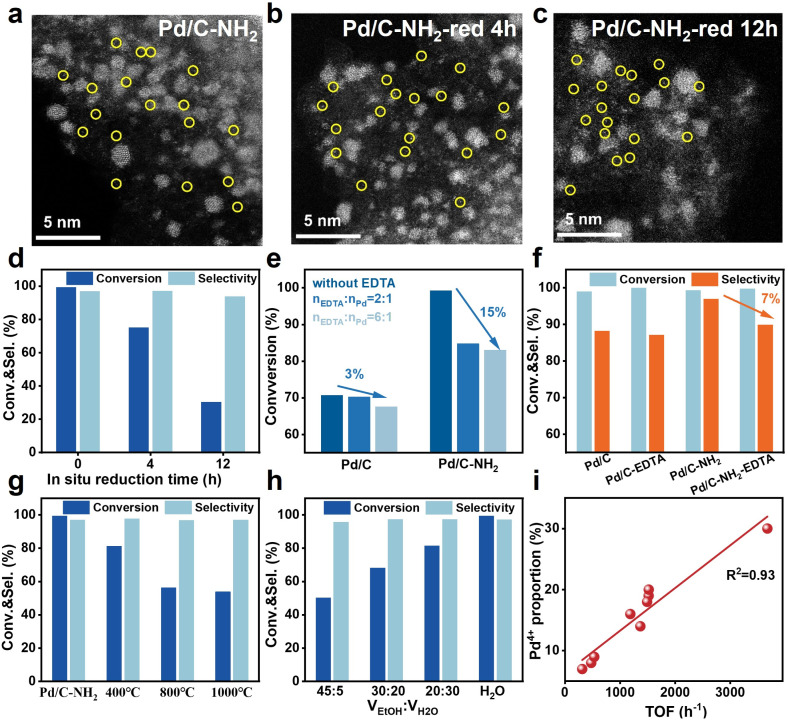
Mechanism of Pd^4+^ on selective hydrogenation of mifepristone. (a–c) AC-STEM images of Pd/C–NH_2_, Pd/C–NH_2_-red 4 h and Pd/C–NH_2_-red 12 h (reduction reaction conditions: 0.5 MPa, 60 °C). (d) Comparison of activity for Pd/C–NH_2_, Pd/C–NH_2_-red 4 h and Pd/C–NH_2_-red 12 h catalysts under identical reaction conditions. (e) Comparison of activity between Pd/C and Pd/C–NH_2_ poisoned with EDTA. (f) Comparison of selectivity for Pd/C and Pd/C–NH_2_ under complete conversion conditions when *n*_EDTA_ : *n*_Pd_ = 6 : 1. (g) Comparison of catalytic performance with supports calculated at different calcination temperatures. (h) Comparison of catalytic performance with different *V*_EtOH_ : *V*_H_2_O_ catalysts for hydrolysis. (i) The linear fitting curve of TOF values *versus* Pd^4+^ content for different catalysts. Reaction conditions: 250 mg mifepristone, 6 mg catalyst, 25 °C, 0.1 MPa H_2_, reaction for 30 min.

To elucidate the specific role of Pd_IV_ SAs in Pd/C–NH_2_ during the hydrogenation of mifepristone, reduction of the catalyst was conducted under relatively harsh conditions (60 °C, 0.5 MPa H_2_, 4 h or 12 h in an autoclave) with the aim of reducing the Pd^4+^ species. AC-STEM images (Fig. 5a–c, S40 and S41) showed that even after reduction, the active Pd species in the catalyst still remained in the form of both SAs and NPs. XPS analysis revealed that the proportion of Pd^4+^ decreased from the original 30% to 13% and finally to 9% (Fig. S42), confirming the successful reduction of Pd^4+^. When the reduced catalysts were applied to the hydrogenation of mifepristone (Fig. 5d, and S43), a significant decrease in catalytic activity was observed under identical conditions. After 12 h of reduction, the activity dropped to 65%. Furthermore, when the reaction time was extended to achieve full conversion of mifepristone, the selectivity decreased from 97% to 93%. To accurately quantify the contribution of Pd_IV_ SA, we systematically adjusted the reaction conditions to compare the selectivity of all catalysts at similar conversion levels.^56^ The experimental results indicate that, although 0.1 wt% Pd/C–NH_2_ required a longer reaction time to achieve comparable conversion, its selectivity (100%, 98%, 98%) at conversion levels of 20%, 50%, and 80% was consistently higher than those of Pd/C–NH_2_ (98%, 97%, 97%) and Pd/C (96%, 94%, 89%) (Fig. S44). This trend further confirms the crucial role of Pd_IV_ SAs in enhancing selectivity.

Ethylenediaminetetraacetic acid (EDTA) and potassium thiocyanate (KSCN) were used as selective poisoning agents to probe the respective contributions of Pd_IV_ SAs and Pd NPs to the reaction.^57,58^ EDTA can selectively poison Pd SA sites, while KSCN can poison both Pd SA and Pd NP sites. As shown in Fig. 5e and S45, when *n*_EDTA_ : *n*_Pd_ = 2 : 1, the conversion of mifepristone over Pd/C–NH_2_ decreased from 99% to 85%, while the selectivity remained largely unchanged. Increasing the ratio to *n*_EDTA_ : *n*_Pd_ = 6 : 1 led to a slight decrease in conversion to 83%. In contrast, for Pd/C, the same poison ratio resulted in only a 3% decrease in activity (from 71% to 68%). When the reaction time was extended to achieve full conversion (Fig. 5f), the selectivity of Pd/C remained almost unchanged, while that of Pd/C–NH_2_ dropped from 97% to 90%. These results indicate that SAs are a key factor in enhancing the selectivity and activity in the selective hydrogenation of mifepristone and further confirm that Pd_IV_ SAs act as active sites. Poisoning experiments with KSCN showed that both Pd/C and Pd/C–NH_2_ catalysts were completely deactivated at *n*_KSCN_ : *n*_Pd_ = 2 : 1, demonstrating that Pd is the sole active site in these catalysts. Subsequently, catalysts obtained through thermal treatment at different temperatures and those modified in different hydrolysis solvents also were also evaluated in the hydrogenation of mifepristone (Fig. 5g, and h). Catalytic activity gradually decreased with increasing thermal calcination temperature, but improved with a higher proportion of deionized water in the hydrolysis solvent. It is noteworthy that a significant linear correlation (*R*^2^ = 0.93) was observed between the TOF values and the proportion of Pd^4+^ species (Fig. 5i and Table S8). This result indicates that the Pd^4+^ content not only modulates the reaction but also exhibits a positive correlation with the TOF, further confirming the critical role of Pd^4+^ species in the catalytic reaction from a kinetic perspective.

### Mechanism analysis

**Fig. 6:**
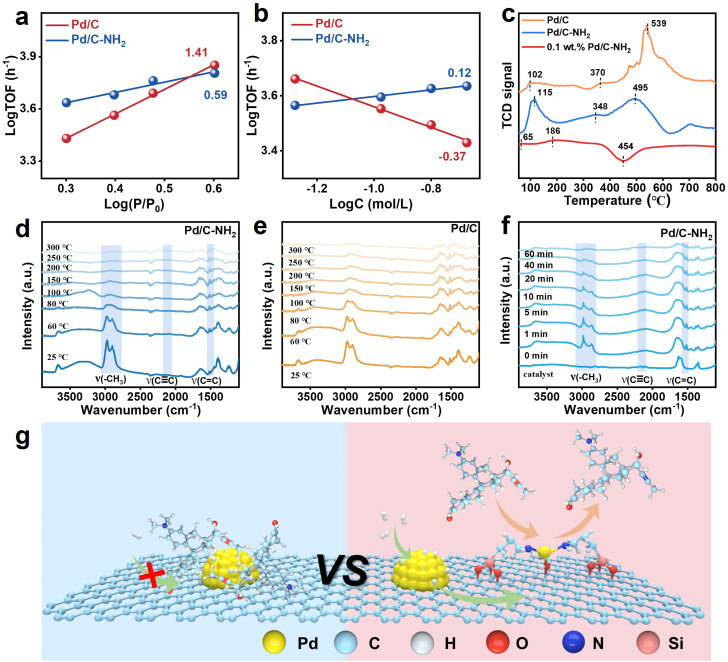
Kinetic studies. (a and b) Kinetic orders with respect to (a) H_2_ and (b) mifepristone over the Pd/C–NH_2_ and Pd/C catalysts. (c) H_2_-TPR of Pd/C, Pd/C–NH_2_ and 0.1 wt% Pd/C–NH_2_. (d and e) *In situ* FT-IR spectra showing the variation of adsorbed mifepristone on Pd/C–NH_2_ and Pd/C with increasing temperature. (f) *In situ* FT-IR spectra showing the variation of adsorbed mifepristone on Pd/C–NH_2_ with H_2_ exposure time. (g) Reaction pathway on the Pd/C and Pd/C–NH_2_ catalysts.

To elucidate the superior activity of Pd/C–NH_2_, the kinetic orders of H_2_ and mifepristone were measured.^28,59,60^ As shown in Fig. 6a, the reaction orders for H_2_ on Pd/C and Pd/C–NH_2_ are 1.41 and 0.59, respectively. The higher order on Pd/C suggests that H_2_ dissociation likely requires multiple adjacent Pd sites, reflecting strong H_2_ pressure dependence. In contrast, the reaction order of 0.59 for Pd/C–NH_2_ implies that H_2_ activation is not hindered by substrate competition. This is attributed to the facilitated dissociation of H_2_ after –NH_2_ modification, reducing its reliance on hydrogen concentration. As shown in Fig. 6b, the reaction orders for mifepristone on Pd/C and Pd/C–NH_2_ are −0.37 and 0.12, respectively. The negative reaction order for Pd/C indicates strong adsorption of the reactant, which covers active sites and may even poison adjacent sites, leading to an overall decrease in activity. The nearly zero reaction order for Pd/C–NH_2_ can be attributed to the presence of Pd_IV_ SAs. These SAs weaken the strong adsorption of mifepristone on Pd NPs, thereby alleviating the competitive adsorption between H_2_ and mifepristone and enhancing hydrogen activation. To verify hydrogen spillover, tungsten oxide (WO_3_) was physically mixed with the catalysts. The yellow WO_3_ turns dark blue upon reaction with spilled hydrogen due to the formation of H_3_WO_3_.^61,62^ After purging under flowing H_2_ for 10 min, both Pd/C–WO_3_ and Pd/C–NH_2_–WO_3_ changed from pale yellow to dark blue (Fig. S46). Notably, Pd/C–NH_2_–WO_3_ exhibited a more pronounced color change to deep green, indicating its stronger hydrogen spillover capability. Hydrogen temperature-programmed reduction (H_2_-TPR) was subsequently performed to further investigate H_2_ adsorption behavior. As shown in Fig. 6c, the low-temperature peaks correspond to the direct reduction of PdO_*x*_,^63,64^ while the high-temperature reduction peaks are attributed to support reduction induced by hydrogen spillover. Pd/C–NH_2_ exhibits the largest peak area at 115 °C, and its support reduction temperature is significantly lowered from 539 °C (Pd/C) to 495 °C, indicating that –NH_2_ modification enhances the low-temperature H_2_ dissociation ability of Pd. For 0.1 wt% Pd/C–NH_2_, the low-temperature reduction peak is relatively weak, suggesting limited H_2_ dissociation capacity. Hydrogen spillover occurs at an early stage and the negative peak at 454 °C likely results from the reverse spillover and recombination release of previously spilled and stored hydrogen,^65^ further confirming that –NH_2_ modification promotes hydrogen spillover. Reactions conducted under H_2_ and D_2_ atmospheres, analyzed by both HPLC and ^1^H NMR, consistently show that all three catalysts exhibit lower reaction rates under D_2_ than under H_2_, with Pd/C–NH_2_ consistently demonstrating the highest activity (Fig. S47, S48 and Table S9). This indicates that Pd NPs are the key hydrogen source determining the overall reaction rate.^59^ Approximately 9% of hydrogenated product (5.6 ppm) was detected in both Pd/C–NH_2_ and Pd/C during deuteration reactions, confirming the participation of water in the reaction.^50^ Notably, Pd/C–NH_2_ yields a higher proportion of deuterated products, indirectly evidencing the occurrence of hydrogen spillover. Catalysts containing SAs exhibit higher hydrogen utilization efficiency and conversion rates within the same reaction time.^66,67^ Although hydrogen spillover also occurs on Pd/C, the absence of suitable acceptors for the activated hydrogen renders the spilled hydrogen incapable of participating in hydrogenation. For 0.1 wt% Pd/C–NH_2_, the scarcity of Pd NPs results in poor H_2_ dissociation ability, making water the primary participant in the initial reaction stage and leading to inferior performance under equivalent conditions. Furthermore, after EDTA poisoning, the H_2_ adsorption capacity (Table S2) of Pd/C–NH_2_ decreases from 301.07 to 111.30 µmol_H_2__ g_Pd_^−1^, correlating with the observed decline in conversion. This confirms the role of Pd_IV_ SAs as critical hydrogen “acceptors”.^52,66^ When these sites are chelated by EDTA, spilled hydrogen cannot be effectively received and utilized, thereby disrupting the entire “activation–spillover–reaction” cycle. These findings further validate the hydrogen spillover mechanism from Pd NPs to Pd SAs. H_2_ dissociation occurs on Pd NPs, and efficient migration and exchange of active hydrogen (effective hydrogen spillover) can only be achieved when both Pd NPs and SAs coexist.

Subsequently, the catalyst was exposed to a certain amount of mifepristone/THF solution, and its desorption behavior was observed under temperature ramping with N_2_ purging. From the spectroscopic data, the characteristic functional groups of mifepristone –CH_3_, C≡C, and C=C are located at 2980, 2862, 2088, and 1520 cm^−1^,^60,68–70^ respectively. Mifepristone desorbed completely from Pd/C–NH_2_ at 200 °C, while on Pd/C, the temperature had to rise to 250 °C or even higher (Fig. 6d, e and S49). On 0.1 wt% Pd/C–NH_2_, desorption occurred at 100 °C, indicating stronger adsorption of mifepristone on Pd/C. Substrate adsorption experiments were conducted to verify this conclusion, and the corresponding XPS results are shown in Fig. S50. The change in binding energy after mifepristone adsorption on Pd/C–NH_2_ (+0.2 eV) was smaller than that on Pd/C (+0.38 eV), indicating a weaker electronic interaction between Pd/C–NH_2_ and mifepristone.^28^ The introduction of SAs weakened the strong adsorption of mifepristone on NPs, thereby providing more active sites for hydrogen dissociation and enhancing catalytic activity. Furthermore, *in situ* infrared spectroscopy was used to monitor changes in mifepristone adsorbed on the catalyst during 25 °C hydrogenation. It was found that on Pd/C–NH_2_, the characteristic peaks of mifepristone weakened and eventually disappeared within 60 min after introducing H_2_. The fundamental reason is that the triple bond in the alkyne side chain was hydrogenated to a double bond, and the propargyl methyl group attached to an sp-hybridized carbon transformed into a common sp^2^-hybridized allyl methyl group, leading to a decrease in the C–H bond vibration frequency. No distinct product peaks were observed because the key new characteristic peaks of the product, aglepristone, overlapped with strong substrate peaks. On Pd/C, this process required 90 min, while on 0.1 wt% Pd/C–NH_2_, the peaks remained largely unchanged even after 120 min (Fig. 6f, and S49), further demonstrating the superior performance of Pd/C–NH_2_.

Based on the above experiments and characterization studies, a reaction mechanism for mifepristone hydrogenation on the catalysts is proposed (Fig. 6g). On Pd/C, H_2_ and the substrate compete for adsorption on Pd NP surfaces. The strong substrate adsorption lowers H_2_ activation efficiency, inhibiting hydrogenation activity and promoting over-hydrogenation. In contrast, Pd/C–NH_2_, which contains both Pd_IV_ SA and Pd NP active sites, enables an alternative hydrogenation pathway. Here, H_2_ is dissociated and activated on Pd NPs, followed by hydrogen spillover to Pd_IV_ SAs, where it reacts with mifepristone adsorbed on the SAs. In this pathway, the weaker adsorption of mifepristone on Pd_IV_ SAs allows the semi-hydrogenated product to desorb readily without over-hydrogenation. This dual-site mechanism^38,52^ contributes to simultaneous enhancement of both activity and selectivity.

## Conclusions

In summary, this study demonstrates a ligand modification strategy for the controlled synthesis of synergistic catalytic centers coexisting Pd_IV_ SAs and Pd NPs, enabling highly efficient and selective hydrogenation of mifepristone. A “grafting-then-coordination” approach facilitated Pd–N/O coordination assisted by both oxygen-containing functional groups and amine ligands on the support surface, leading to the stabilization of Pd_IV_ SAs. Under mild conditions, the Pd/C–NH_2_ catalyst exhibited outstanding catalytic performance, achieving 99% conversion and 97% selectivity in the selective hydrogenation of mifepristone, significantly surpassing Pd/C. The electron-deficient nature of Pd_IV_ SAs mitigates substrate poisoning on Pd NP sites, thereby enhancing H_2_ activation. Concurrently, H_2_ activated on Pd NPs undergoes spillover to the hydrogen-deficient Pd_IV_ SAs, promoting hydrogenation of mifepristone adsorbed on the Pd_IV_ SA sites. The present ligand modification strategy enables the formation of Pd_IV_ SAs that transform conventional poisoning sites into productive active centers. The collaborative effect between Pd_IV_ SAs and Pd NPs provides a novel pathway for the selective hydrogenation of mifepristone and offers valuable insights for the rational design of catalysts for steroidal drug synthesis.

## Author contributions

M. J. major experimental production, formal analysis, methodology, investigation, data curation, writing – original draft. Y. L. experimental production, methodology, visualization. X. L., Z. X. Y., C. M. C. and Y. M. H. validation, formal analysis, data curation, writing – review & editing. F. J. S., X. N. L., J. X. H. and S. D. visualization, writing – review & editing. Z. Z. W: methodology, conceptualization, resources, supervision, project administration, writing – review & editing. J. G. W: methodology, funding acquisition, resources, supervision, project administration, writing – review & editing.

## Conflicts of interest

The authors declare no competing financial interests.

## Supplementary Material

SC-017-D5SC08632A-s001

## Data Availability

The authors confirm that the data supporting the findings of this study are available within the article and its supplementary information (SI). The data supporting this article have been included as part of the supporting information. Supplementary information: chemicals, catalyst evaluation and analysis and catalyst characterization. Characterization data, NMR spectra, HPLC traces, and additional figures supporting the results presented in the main text. See DOI: https://doi.org/10.1039/d5sc08632a.
