# Supplementary material for: Beyond the Lindlar catalyst: highly-oxidized Pd single atoms as promoters for alkyne semi-hydrogenation
Source: Chem Sci. 2026 Jan 6;17(9):4780–93. doi: 10.1039/d5sc08632a (PMC12809463; doi:10.1039/d5sc08632a)
Supplement: SC-017-D5SC08632A-s001 [file SC-017-D5SC08632A-s001.pdf]

Supplementary Information (SI) for Chemical Science.
This journal is © The Royal Society of Chemistry 2026

Beyond Lindlar Catalyst: Highly-oxidized Pd Single Atoms as Promoter for Alkyne Semi-hydrogenation

Ming Jiang[a ‡], Yao Lv[b ‡], Zhongzhe Wei[a], Xu Liu[a],1 Zhixiang Yang[a], Chuanming Chen[a], Yiming Hu[a], Fangjun Shao[a], Xiaonian Li[a], Jiaxing Hu*[c], Sheng Dai*[b], Jianguo Wang*[a]*

[a.] Institute of Industrial Catalysis, College of Chemical Engineering, Zhejiang University of Technology, Hangzhou 310032, P. R. China.

[b.] Key Laboratory for Advanced Materials and Feringa Nobel Prize Scientist Joint Research Center, School of Chemistry and Molecular Engineering, East China University of Science & Technology, Shanghai 200237, P. R. China.

[c.] Zhejiang Huahai Pharmaceutical Co., Ltd, Linhai 317024, P. R. China.

[*]Corresponding author. weizhzhe@zjut.edu.cn; hujiaxing@huahaipharm.com; shengdai@ecust.edu.cn; jgw@zjut.edu.cn.

Experimental Section

1. Chemicals

3-aminopropyltriethoxysilane (APTES/KH-550, AR), potassium thiocyanate (KSCN, AR), and tungsten trioxide (WO_3, AR) were purchased from Macklin Inc. Potassium tetrachloropalladate (K_2PdCl_4, AR) and Lindlar catalyst (BD00818184, palladium supported on calcium carbonate, poisoned with lead, palladium loading 5.04 wt.%) were obtained from Bide Pharmatech Co., Ltd. 3-aminopropyltrimethoxysilane (KH-540, AR) and n-propyltriethoxysilane (KH-330, AR) were sourced from Energy Chemical. Ethylenediaminetetraacetic acid (EDTA, AR), sodium borohydride ($NaBH_4$, AR), and anhydrous ethanol were procured from Sinopharm Chemical Reagent Co., Ltd. [N-(3-Methoxy) propyl] ethylenediamine (KH-792, AR), anilinomethyltriethoxysilane (SJ-42, AR), methyltrimethoxysilane (D-20, AR), and mifepristone were supplied by Aladdin. 3-glycidyloxypropyltrimethoxysilane (KH-560, AR), tetrahydrofuran (THF, AR), and n-butylamine were purchased from Tansoole. Ureidopropyltriethoxysilane (A-1160, AR) was acquired from Meryer. Commercial Pd/C catalyst (AUP53261, 55% water content, 5 wt.% Pd) was obtained from Auonsi Reagents. Activated carbon was provided by Shanghai Xing Chang Activated Carbon Co., Ltd (lot number 2384017). All reagents were used as received without further purification. Gases (H_2 and Ar) used for catalyst synthesis and catalytic reactions were of ultra-high purity.

2. Catalyst evaluation and analysis

In the semi-hydrogenation reaction system of mifepristone, the catalyst (6 mg) and mifepristone (250 mg) were thoroughly dispersed in 10 mL of THF and 1 mL of deionized water. The mixture was placed in a 100 mL batch autoclave reactor, purged with H_2 more than five times to remove air, and then reacted at 25 °C under 0.1 MPa H_2 pressure with stirring at 800 rpm. During the reaction, aliquots of the reaction mixture were withdrawn using a syringe, filtered, diluted with anhydrous ethanol, and analyzed by high-performance liquid chromatography using a mobile phase of acetonitrile/water (6:4, v/v). In this study, the selectivity of all catalysts is defined as the ratio of the target product aglepristone (the cis-olefin, whose reaction equation is shown in Figure 4d)

relative to all hydrogenation products. Replicate experiments conducted under identical reaction conditions showed that both conversion and selectivity values were reproducible within ± 2%. Based on the original experimental procedure, the reaction scale was increased by a factor of four. A mixture of 24 mg catalyst and 1000 mg substrate was dispersed in 10 mL THF and 1 mL deionized water, then stirred (800 rpm) at 25 °C under 0.1 MPa H_2 atmosphere. After 30 min of reaction, the process was temporarily stopped. A small aliquot of the reaction mixture was withdrawn, filtered, diluted, and analyzed by high-performance liquid chromatography. The remaining solution was vacuum-filtered and thoroughly washed with anhydrous ethanol. The recovered catalyst was collected and dried overnight at 70 °C under vacuum for subsequent characterization and recycling tests. In the recycling experiments, the catalyst was replenished to its initial loading amount, and the reaction was repeated under identical conditions to evaluate the catalytic performance. To accurately evaluate the intrinsic catalytic activity of the catalyst in the semi-hydrogenation of mifepristone, the TOF was calculated based on the reaction rate at low conversion (< 30%) using the following formula:

$$TOF(h^{-1}) = \frac{Conv.(\%) \times n}{\left(\frac{m(mg) \times wt\% \times D_m}{M}\right)} \div \left(\frac{T(min)}{60}\right)$$

where Conv. (%) represents the conversion of mifepristone, n is the initial moles of mifepristone, M is the molar mass of Pd, m is the mass of catalyst, wt. (%) is the loading of Pd, T is the reaction time (h), and D_m is the dispersion of Pd species (determined by H_2-O_2 titration).

3. Catalyst characterization

The microstructure and elemental distribution of catalysts were observed using a transmission electron microscope (TEM, Tecnai G2F30S-Twin) and high-resolution transmission electron microscopy (HRTEM) combined with energy dispersive X-ray spectroscopy(EDS, Xplore80) with an operating voltage of 300 kV. Aberration-corrected scanning transmission electron microscopy (AC-STEM) operated on a ThermoFisher Themis Z transmission electron microscope equipped with two aberration correctors. High angle annular dark-field (HAADF)-STEM images were recorded using a convergence semi-angle of 25 mrad, and inner- and outer collection angles of 47 and 200 mrad, respectively. Energy-dispersive X-ray spectroscopy (EDS) was carried out using 4 in-column Super-X detectors. EELS data was acquired using a Gatan Enfinium ER (model 977)

EELS spectrometer with a dual EELS function. The N_2 adsorption-desorption measurements were conducted at -196 °C using a surface characterization analyzer (ASAP 2460). Then, the specific surface area and average pore size of the catalysts were determined using the Brunauer-Emmett-Teller (BET) method and the formula 4V/A, respectively. The total pore volume was evaluated based on the Barrett-Joyner-Halenda (BJH) model. The crystal structure of the samples was analyzed by X-ray diffraction (XRD, PANalytical X, pert PRO) with Cu-K_{α} radiation (λ = 0.1541 nm), operating at 40 kV and 40 mA. Raman spectra were acquired using a HORIBA HR Evolution system with a 532 nm laser source. Fourier transform infrared (FT-IR) spectrometer employed the Nicolet iS50 from Thermo-Fisher Scientific, the potassium bromide and catalyst were ground homogeneously and pressed into tablets, which were fed into the infrared spectrometer and scanned over a wavelength range of 4000-400 cm^{-1} with a scanning frequency of 32 times. CO diffuse reflectance infrared Fourier transform spectroscopy (CO-DRIFTS) measurements were performed on a Thermo Scientific Nicolet IS 50 spectrometers equipped with an in situ cell (XF808-18R, developed by the Dalian Institute of Chemical Physics, Chinese Academy of Sciences) and an MCT detector. Prior to measurement, the catalyst sample was pretreated in N_2 flow (20 $mL\cdot min^{-1}$) at 200 °C for 1 h, followed by cooling to 30 °C to record the background spectrum. Afterward, CO was introduced into the sample at a flow rate of 20 $mL\cdot min^{-1}$ until adsorption saturation, followed by N_2 purging to remove physisorbed gaseous CO. Finally, the CO-DRIFTS spectrum was collected. In situ FT-IR spectroscopy for substrate adsorption was conducted on the same spectrometer using an in situ transmission cell equipped with an MCT detector. The catalyst was first pretreated in N_2 flow (20 $mL\cdot min^{-1}$) for 0.5 h, and its background spectrum was recorded. Subsequently, the substrate was dropped onto the catalyst, and the temperature was increased at 5 $°C\cdot min^{-1}$under N_2 flow. After reaching a specified temperature, the system was stabilized for 10 min, and spectra were collected at different temperatures until the substrate was nearly completely desorbed, after which the gas flow was stopped. In a separate experiment, after pretreatment of the catalyst in N_2 flow (20 $mL\cdot min^{-1}$) for 0.5 h and recording of the background spectrum, the substrate was dropped onto the catalyst. H_2 was then introduced at a flow rate of 20 $mL\cdot min^{-1}$ until the substrate was largely desorbed, and spectra were collected at different purging times. Pd K-edge analysis was performed with Si (111) crystal monochromators at the BL14W1 beamlines at the Shanghai Synchrotron Radiation Facility (SSRF) (Shanghai, China). Before the

analysis at the beamline, samples were pressed into thin sheets with 1 cm in diameter and sealed using Kapton tape film. The XAFS spectra were recorded at room temperature using a 4-channel Silicon Drift Detector (SDD) Bruker 5040. Pd K-edge extended X-ray absorption fine structure (EXAFS) spectra were recorded in transmission mode. Negligible changes in the line-shape and peak position of Pd K-edge XANES spectra were observed between two scans taken for a specific sample. The XAFS spectra of these standard samples (Pd and PdO) were recorded in transmission mode. The spectra were processed and analyzed by the software codes Athena and Artemis. X-ray photoelectron spectroscopy (XPS) modeled as Thermo Scientific K-Alpha was utilized to investigate the elemental composition and chemical state on the catalyst surface, and the charge correction of C, N, O, Si and Pd elements was performed with a carbon standard peak (284.8 eV). The actual Pd loading was determined by inductively coupled plasma optical emission spectrometry (ICP-OES, Agilent 720ES) after digesting the sample in a mixture of hydrochloric acid and nitric acid (volume ratio 3:1). The dispersion of metallic Pd was measured by hydrogen-oxygen titration (H_2-O_2) using an Auto Chem II 2920 apparatus. Hydrogen Temperature-Programmed Reduction (H_2-TPR) was performed by Microtrac BELCat II: the sample was dried and treated and then fed with 10 % H_2/Ar mixture (30 - 50 mL/min) for 1 h until saturation, and the Ar gas stream (30 - 50 mL/min) was switched to purge for 1 h to remove the weakly physically adsorbed H_2 on the surface, and finally the sample was desorbed at a temperature increase rate of 10 °C/min to 800 °C under Ar atmosphere, and the desorbed gas was detected by thermal conductivity detector (TCD). ^{1}H nuclear magnetic resonance (^{1}H NMR) spectroscopy analyses were performed on a AVANCE NEO 400 M equipment. Following the general evaporation purification procedure, the corresponding product was obtained as a pale yellow solid, using deuterated chloroform as the solvent, with piperazine added as the internal standard.

Supporting Figures and Tables

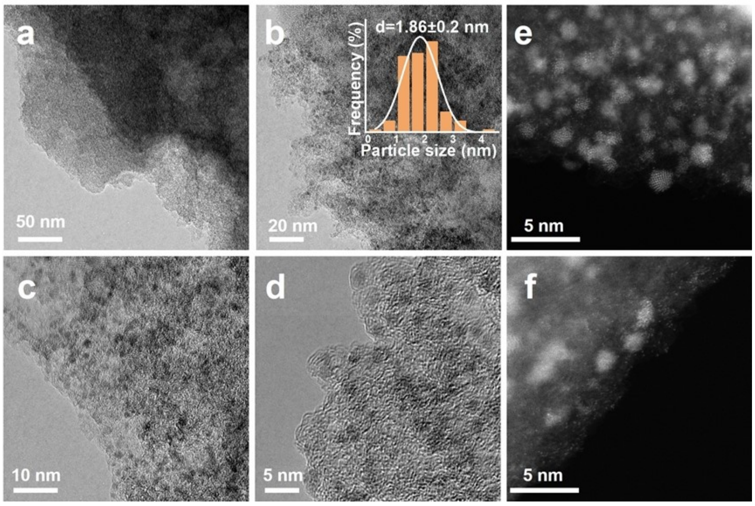

Figure S1. (a-c) TEM image of Pd/C-NH_2. (d) HRTEM images of Pd/C-NH_2. (e-f) AC-STEM images of Pd/C-NH_2.

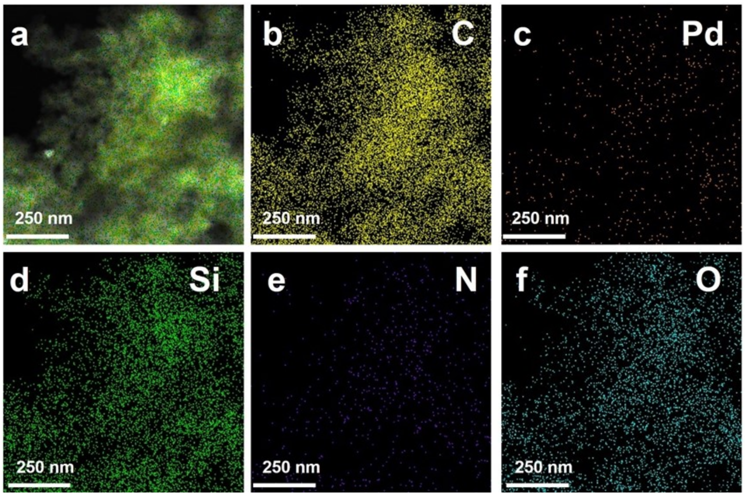

Figure S2. EDS elemental mapping of Pd/C-NH_2.

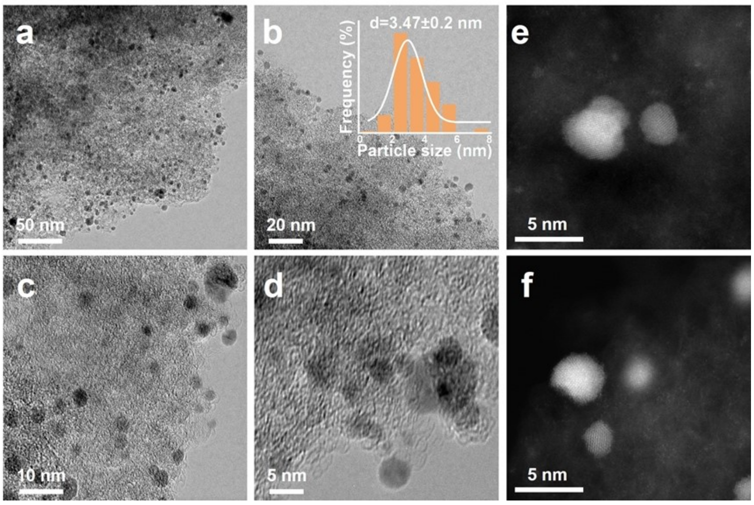

Figure S3. (a-c) TEM image of Pd/C. (d) HRTEM images of Pd/C. (e-f) AC-STEM images of Pd/C.

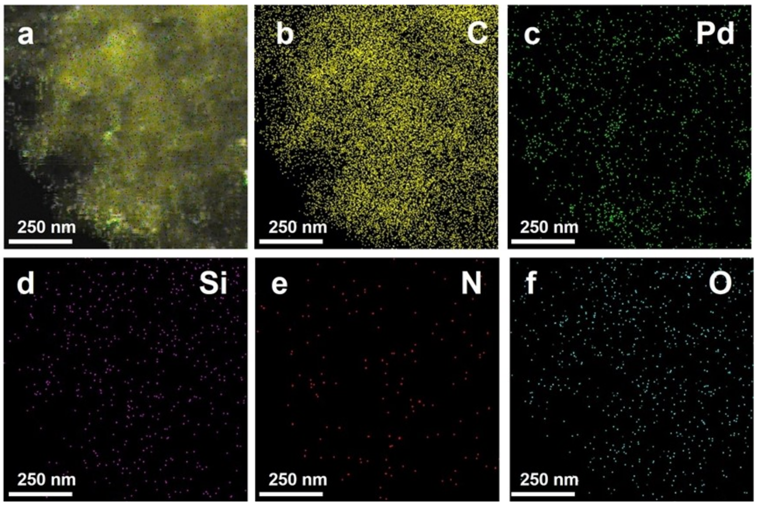

Figure S4. EDS elemental mapping of Pd/C.

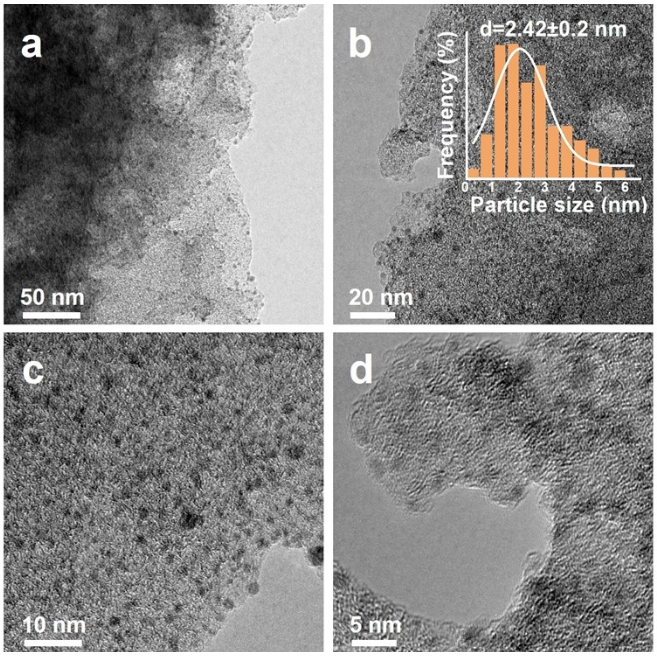

Figure S5. (a-c) TEM images and particle size distribution of Pd/C-NH_2-5h. (d) HRTEM images of Pd/C-NH_2-5h.

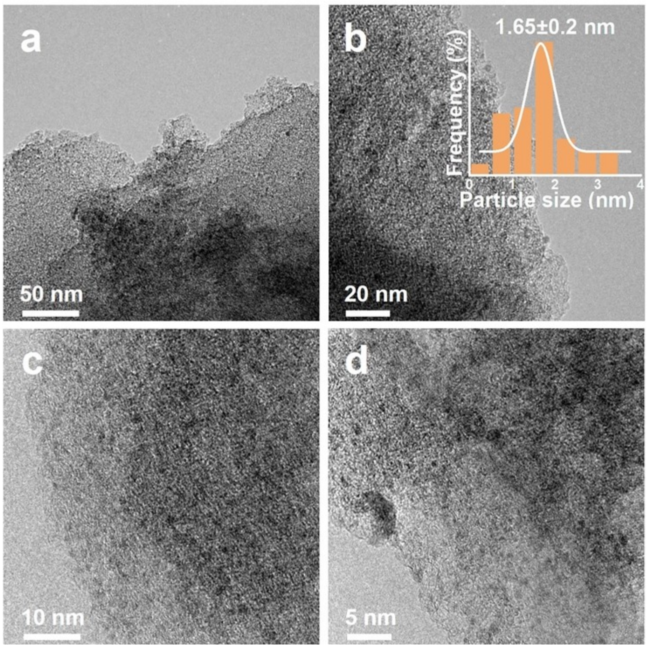

Figure S6. (a-c) TEM images and particle size distribution of Pd/C-NH_2-24h. (d) HRTEM images of Pd/C-NH_2-24h.

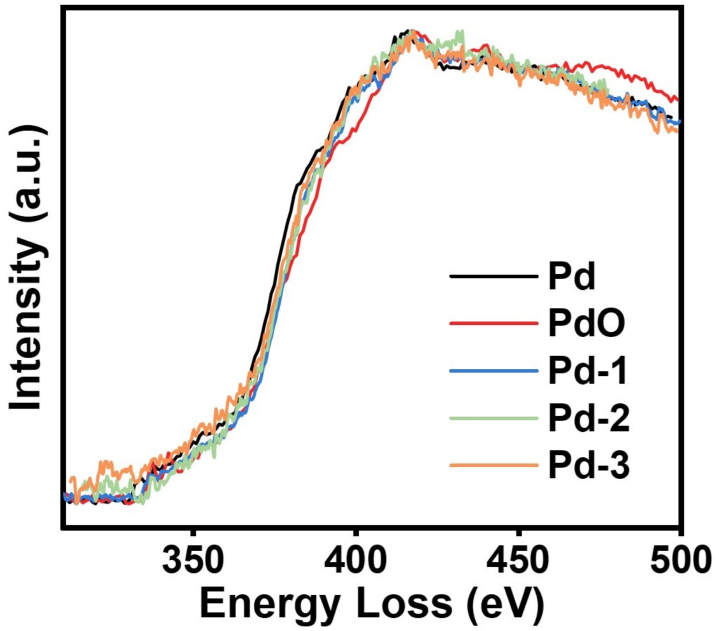

Figure S7. EELS spectra characterizing the region in the image.

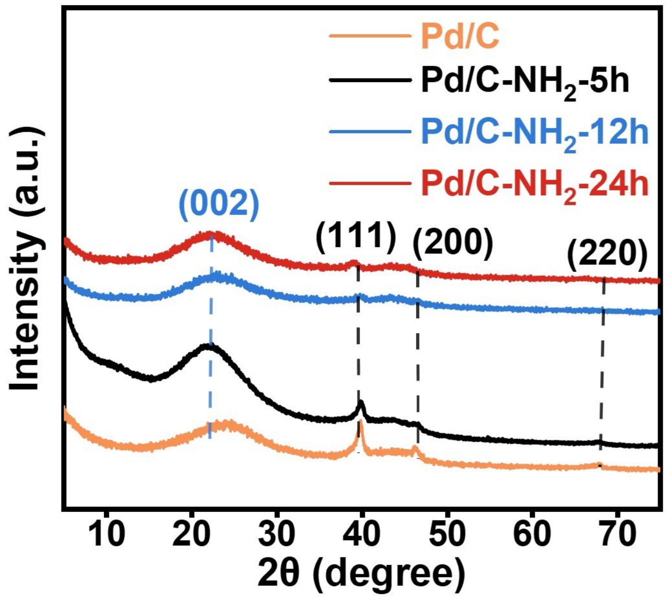

Figure S8. XRD patterns of Pd/C and Pd/C-t.

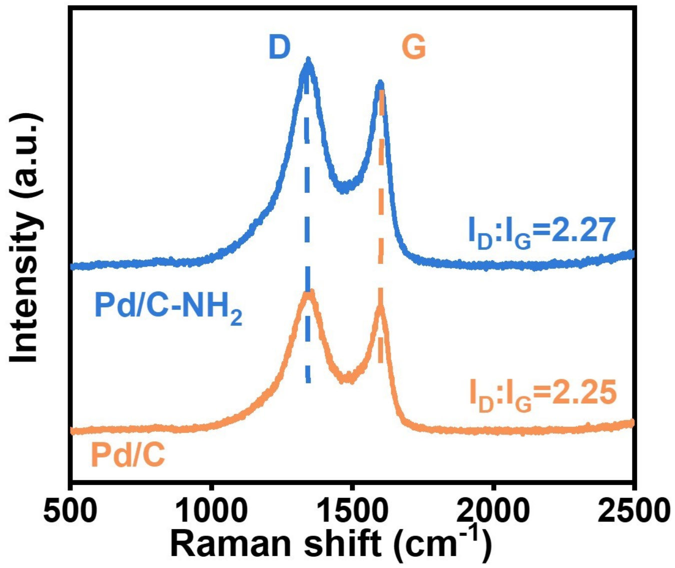

Figure S9. Raman patterns of Pd/C and Pd/C-NH_2.

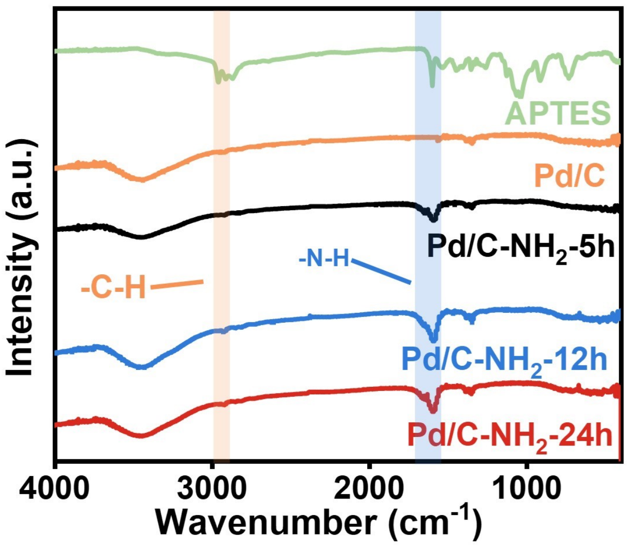

Figure S10. FTIR patterns of Pd/C and Pd/C-t.

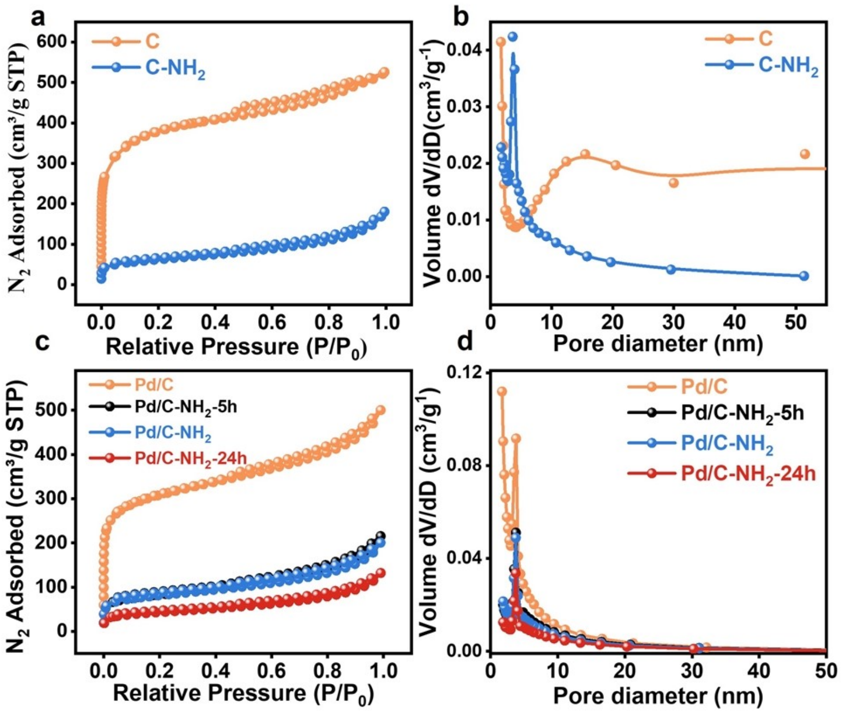

Figure S11. N_2 adsorption-desorption isotherm of (a, b) C and C-NH_2, (c, d) Pd/C and Pd/C-NH_2-t.

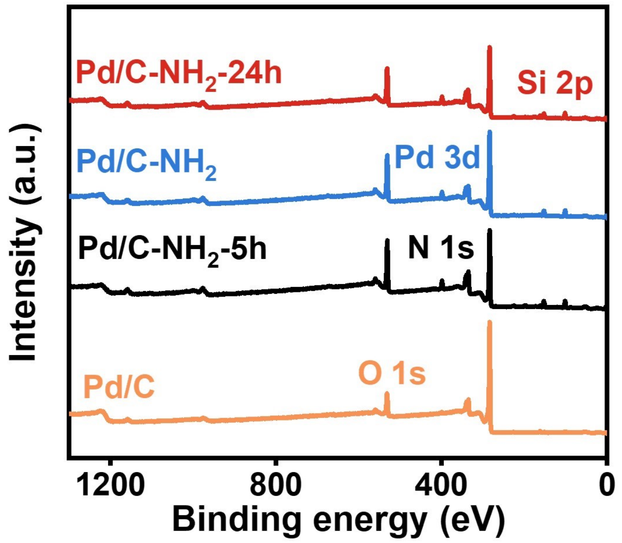

Figure S12. The survey XPS spectra of APTES modified time series catalysts.

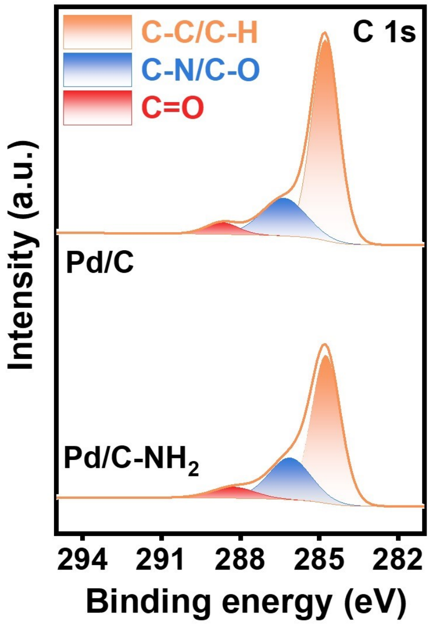

Figure S13. High-resolution C 1s XPS spectra of Pd/C and Pd/C-NH_2.

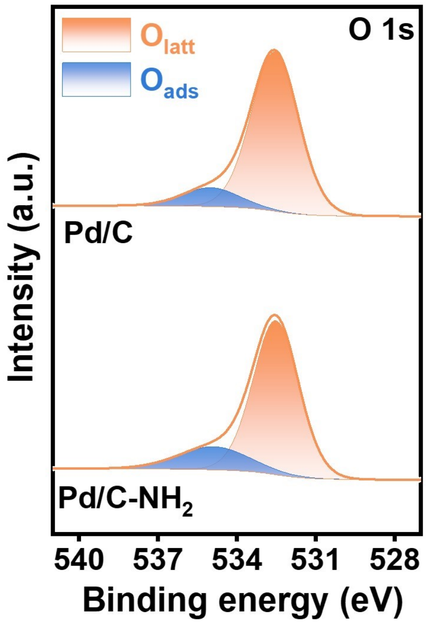

Figure S14. High-resolution O 1s XPS spectra of Pd/C and Pd/C-NH_2.

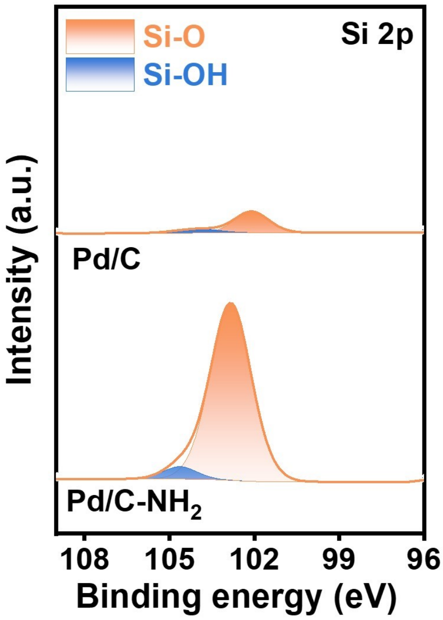

Figure S15. High-resolution Si 2p XPS spectra of Pd/C and Pd/C-NH_2.

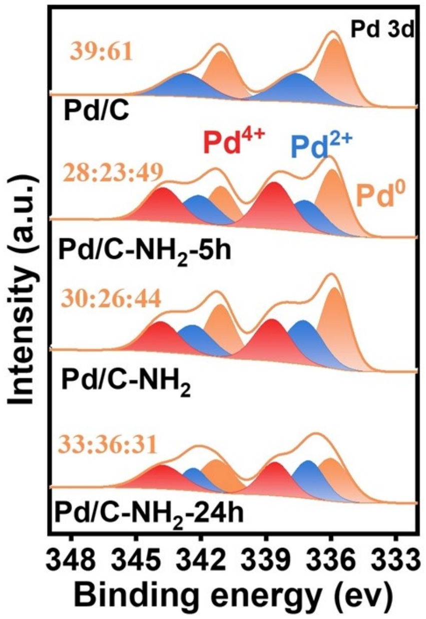

Figure S16. High-resolution Pd 3d XPS spectra of Pd/C and Pd/C-NH_2-t.

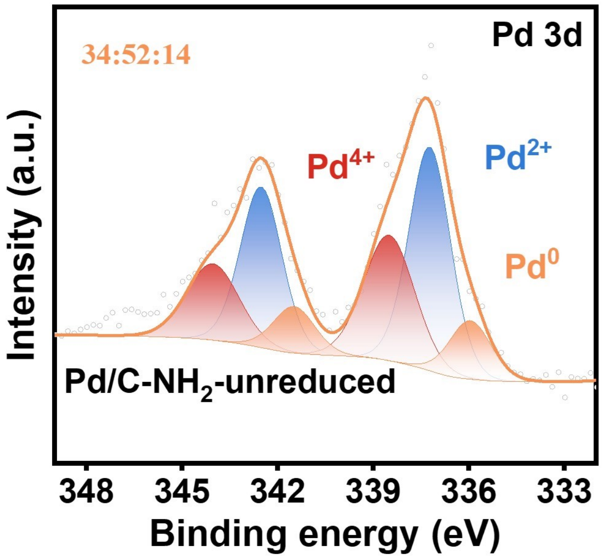

Figure S17. High-resolution Pd 3d XPS spectra of Pd/C-NH_2-unreduced was not reduced by $NaBH_4$.

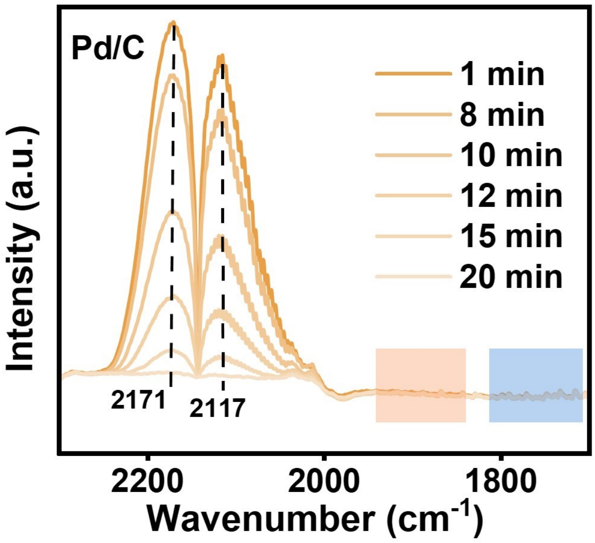

Figure S18. In situ DRIFT spectra of CO chemisorption on Pd/C.

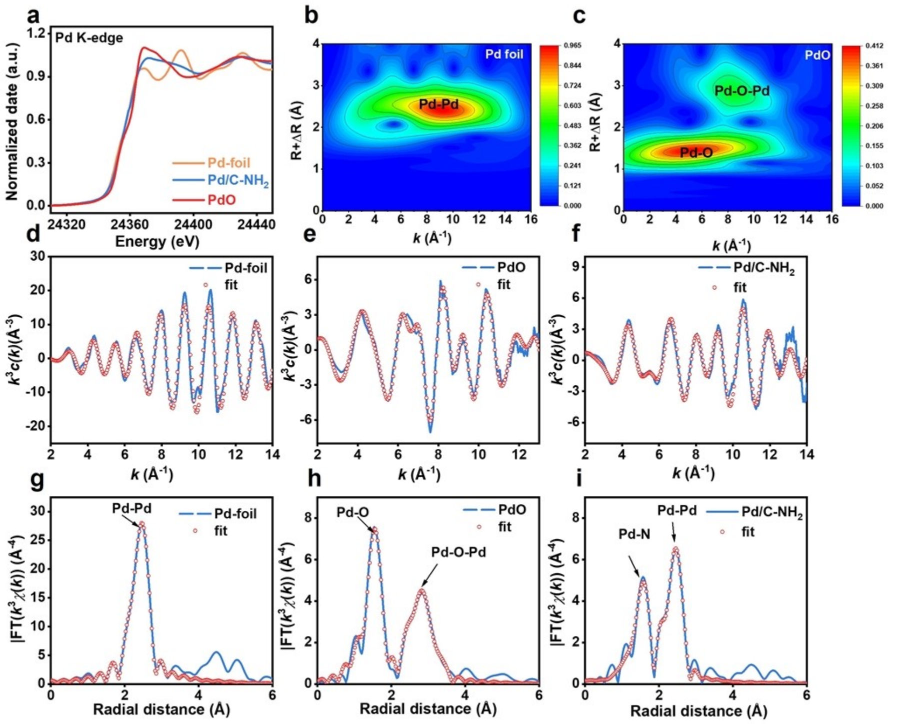

Figure S19. XAFS analysis of Pd/C-NH_2. (a) Pd K-edge XANES spectra Pd/C-NH_2. Pd foil and PdO were applied for comparison. (b, c) WT-EXAFS plots of Pd and PdO. (d-f) R-spaced FT-EXAFS of Pd K-edge. (g-i) space.

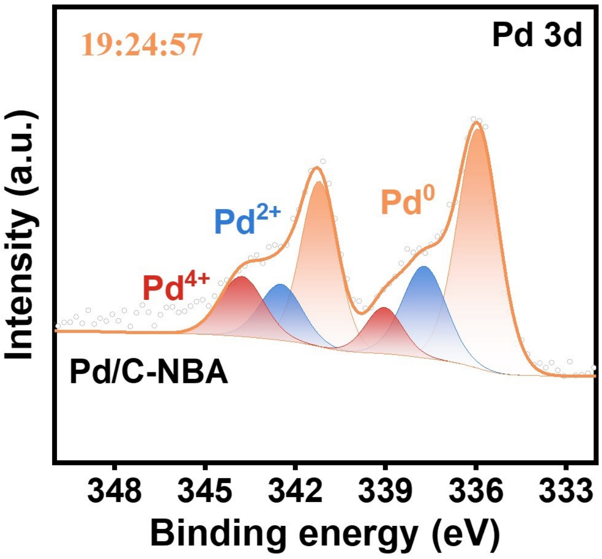

Figure S20. High-resolution Pd 3d XPS spectra of Pd/C-NBA catalysts.

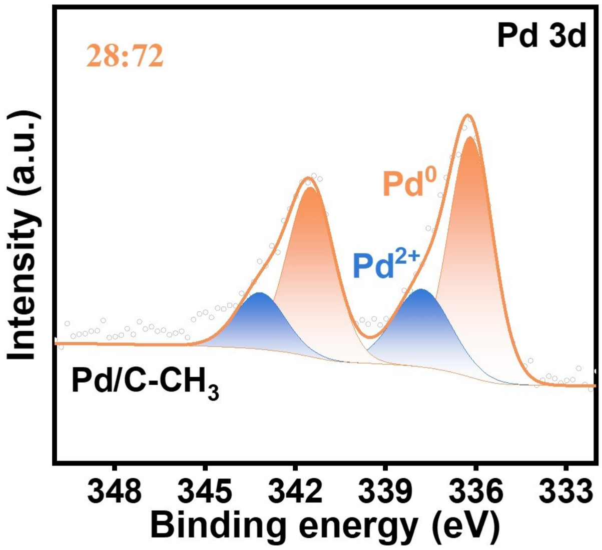

Figure S21. High-resolution Pd 3d XPS spectra of Pd/C-CH_3 catalysts.

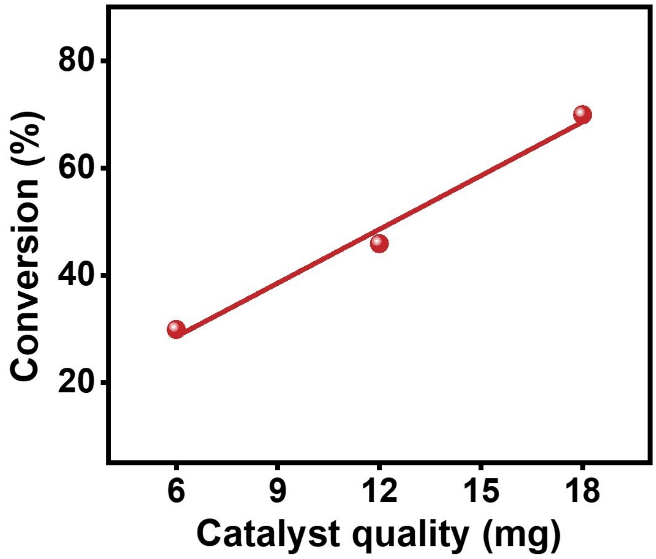

Figure S22. The conversion curves versus catalyst quality over Pd/C-NH_2 under identical conditions.

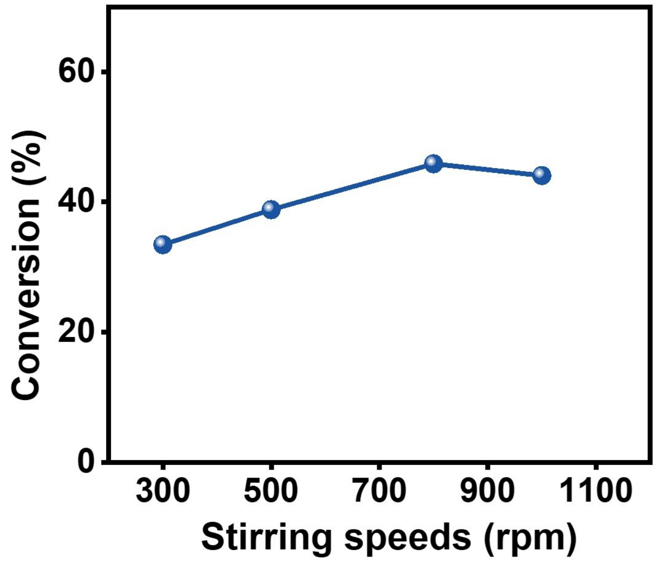

Figure S23. The conversion curves versus stirring speed over Pd/C-NH_2 under identical conditions.

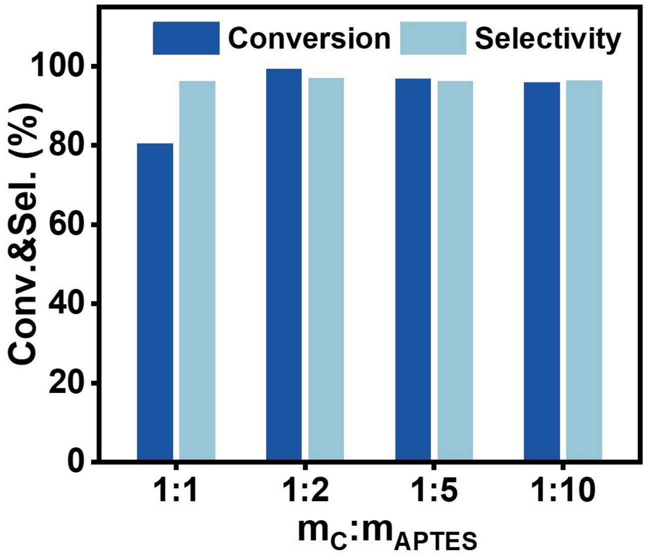

Figure S24. Under the condition that other preparation conditions remain unchanged, the influence of changing the mass ratio of C to APTES on catalytic performance was studied.

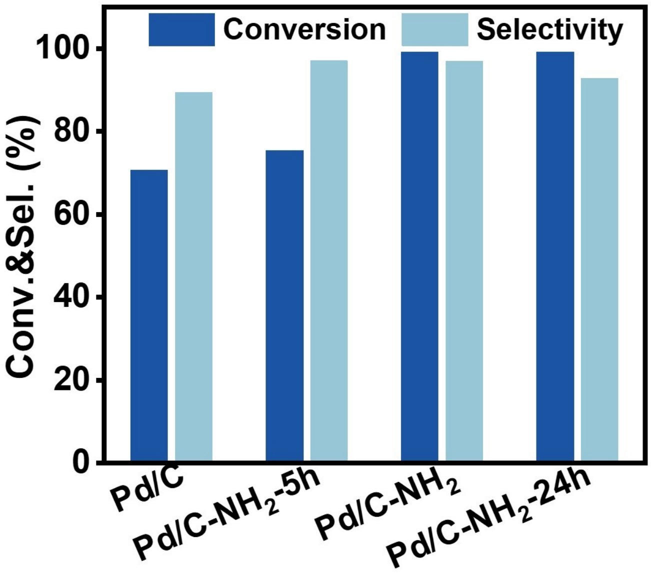

Figure S25. Under the same preparation conditions, the effects of different stirring times on catalytic performance were studied when the mass ratio of C to APTES was changed to 1:2.

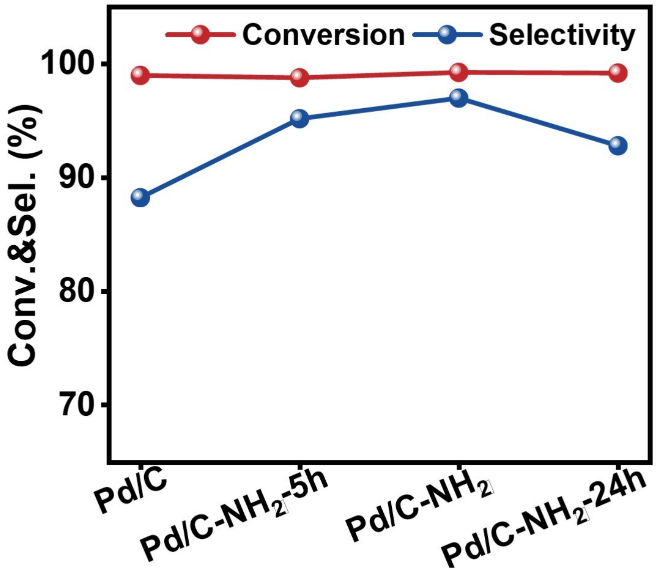

Figure S26. The selectivity differences under varying modification times at near-complete conversion conditions.

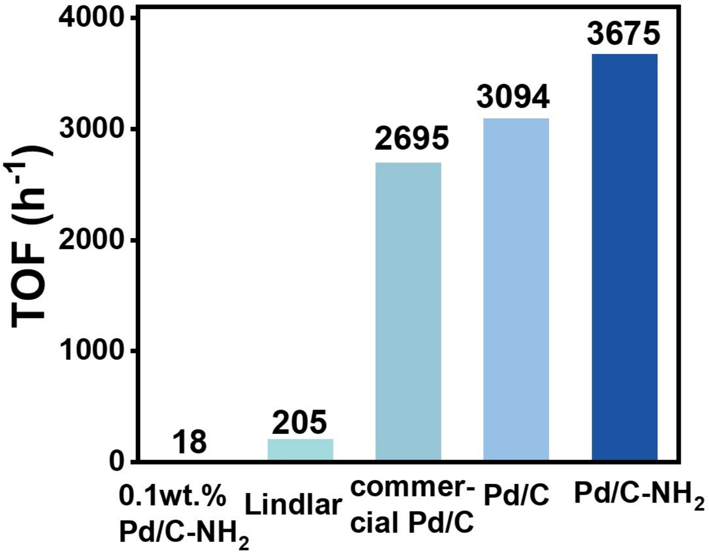

Figure S27. TOF value of 0.1wt.%Pd/C-NH_2, Lindlar catalyst, commercial Pd/C, Pd/C and Pd/C-NH_2 catalysts.

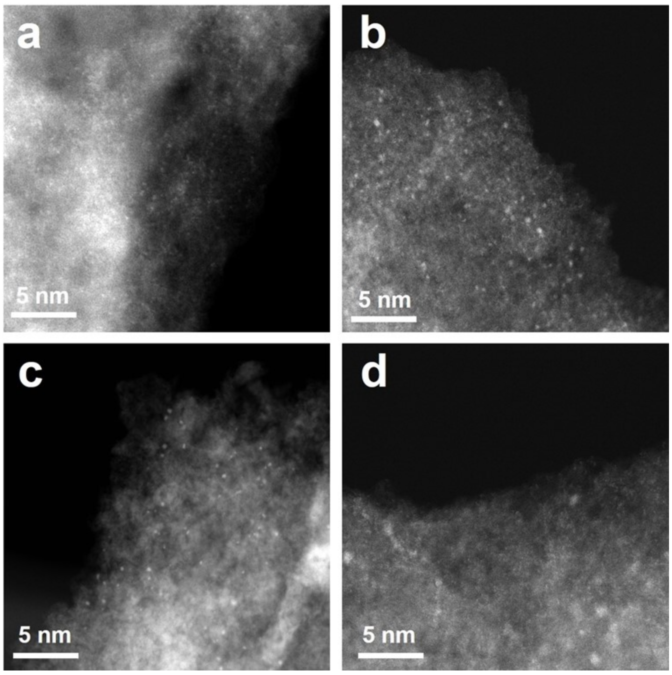

Figure S28. AC-STEM images of Catalyst 0.1wt.%Pd/C-NH_2.

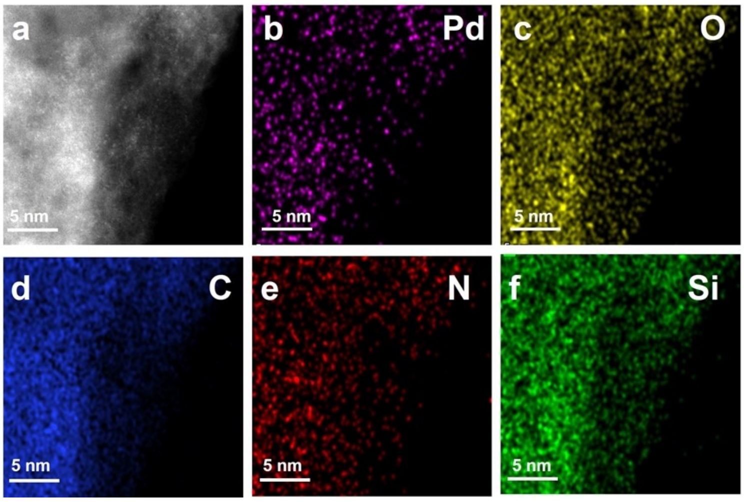

Figure S29. EDS elemental mapping of 0.1wt.%Pd/C-NH_2.

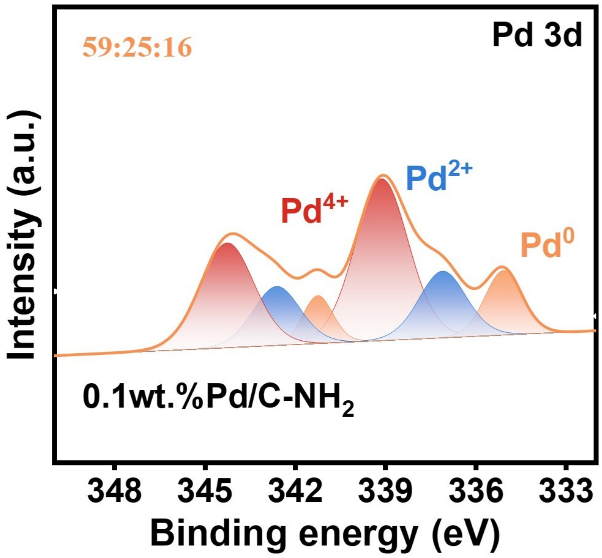

Figure S30. High-resolution Pd 3d XPS spectra of 0.1wt.%Pd/C-NH_2 catalysts.

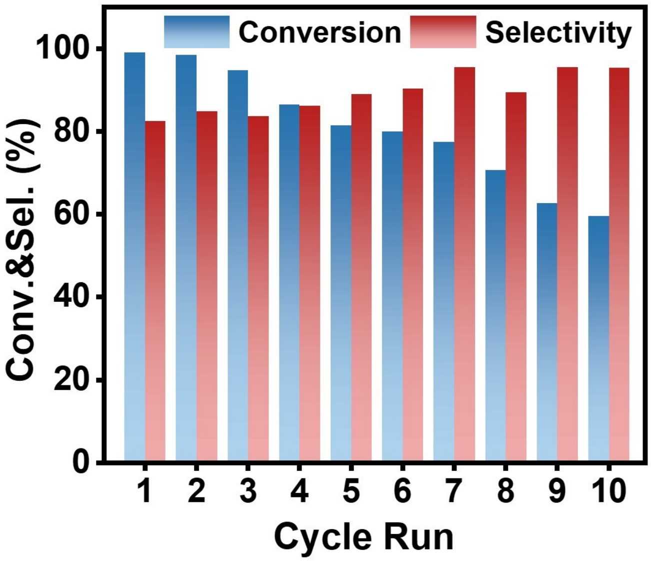

Figure S31. Stability cyclic test of Pd/C.

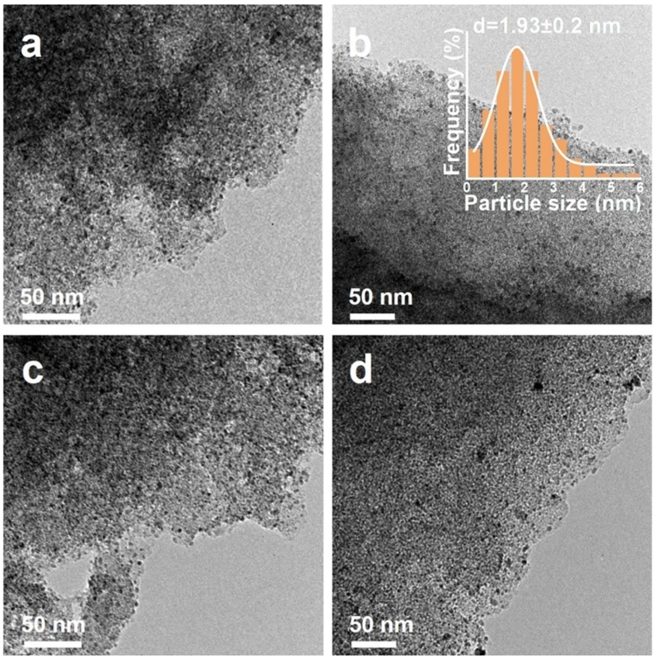

Figure S32. TEM images of Pd/C-NH_2 after ten cycles.

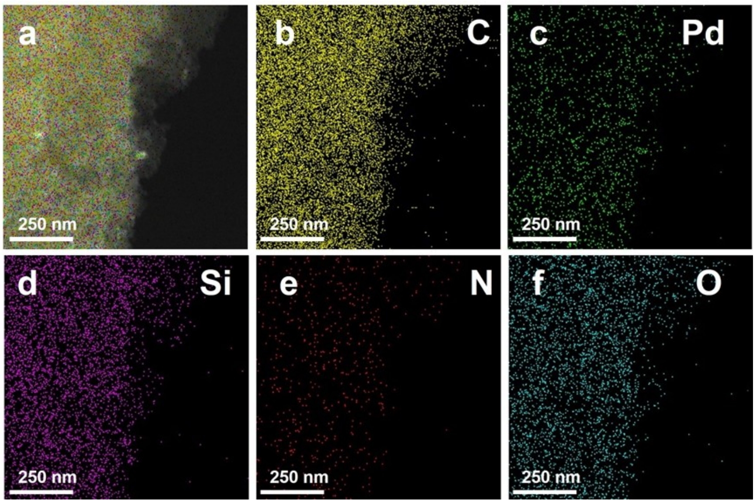

Figure S33. EDS elemental mapping of Pd/C-NH_2 after ten cycles.

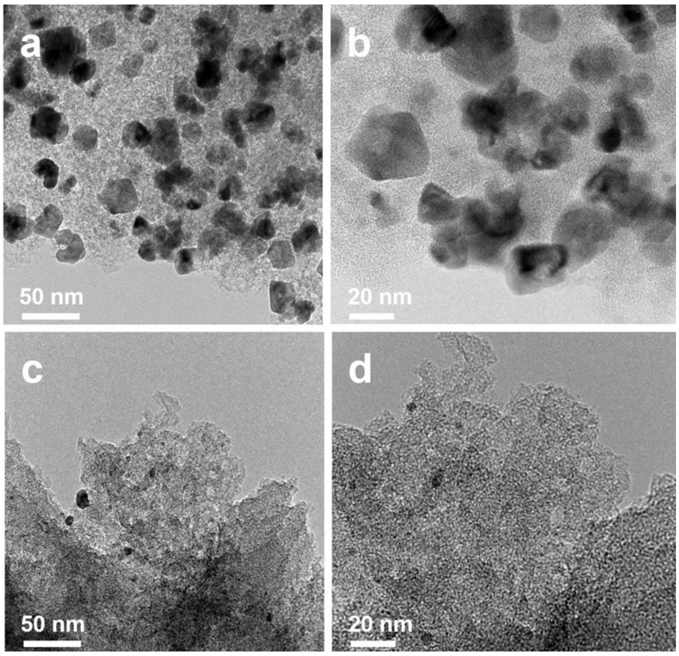

Figure S34. TEM images of Pd/C after ten cycles.

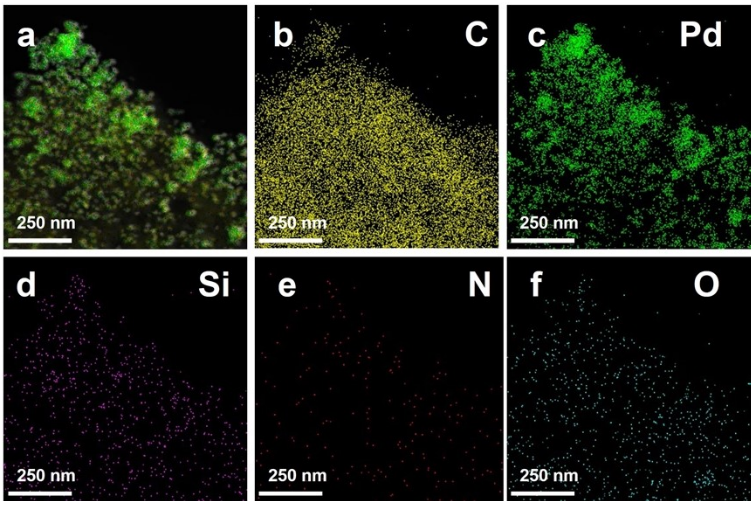

Figure S35. EDS elemental mapping of Pd/C after ten cycles.

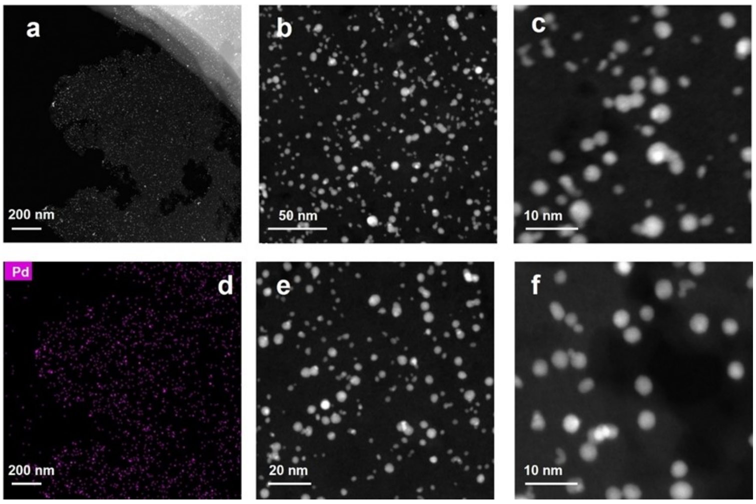

Figure S36. HAADF-STEM images of Pd/C-NH_2 after ten cycles.

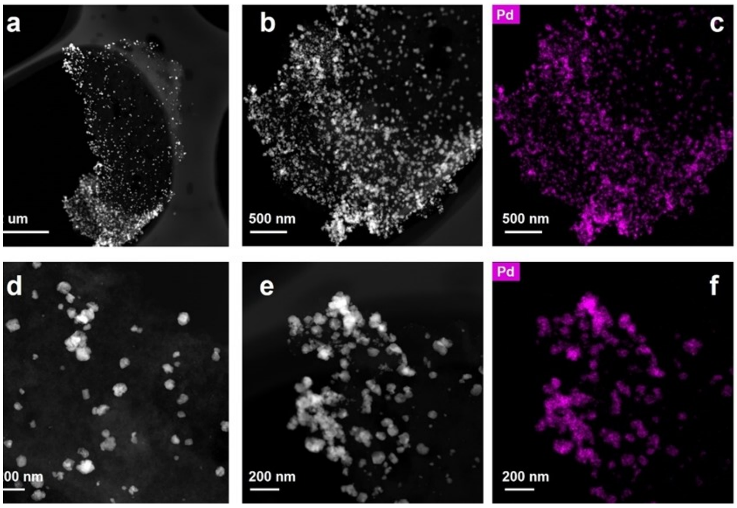

Figure S37. HAADF-STEM images of Pd/C after ten cycles.

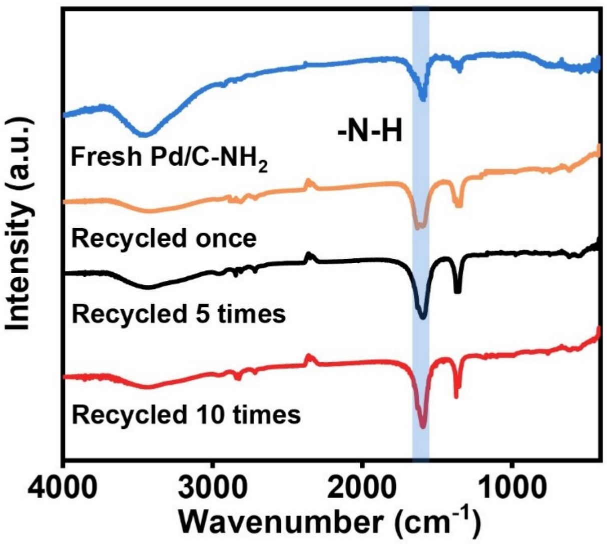

Figure S38. FTIR patterns of Pd/C-NH_2 after recycling tests.

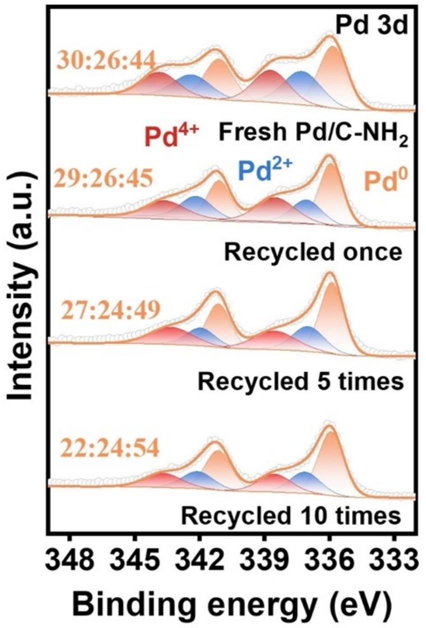

Figure S39. High-resolution Pd 3d XPS spectra of Pd/C-NH_2 after recycling tests.

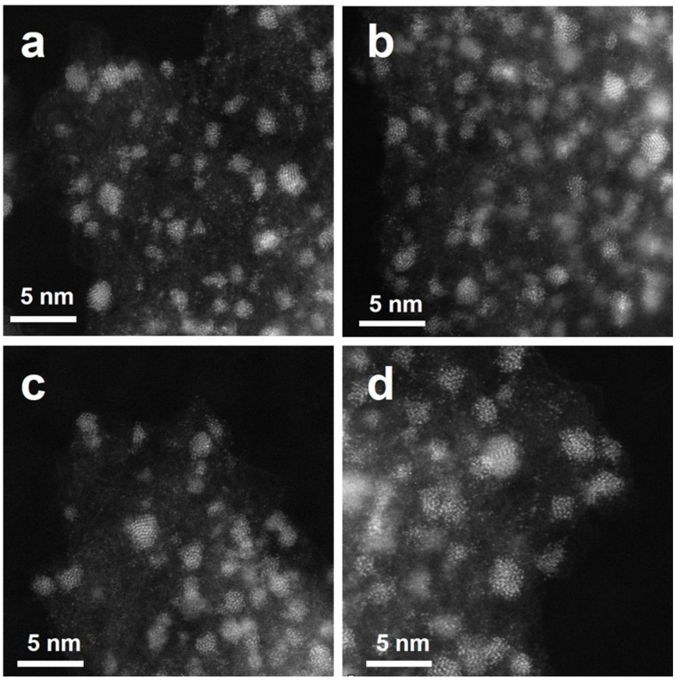

Figure S40. AC-STEM images of Catalyst Pd/C-NH_2-red 4h was synthesized via in-situ hydrogen reduction for 4 h (0.5 MPa, 60 °C).

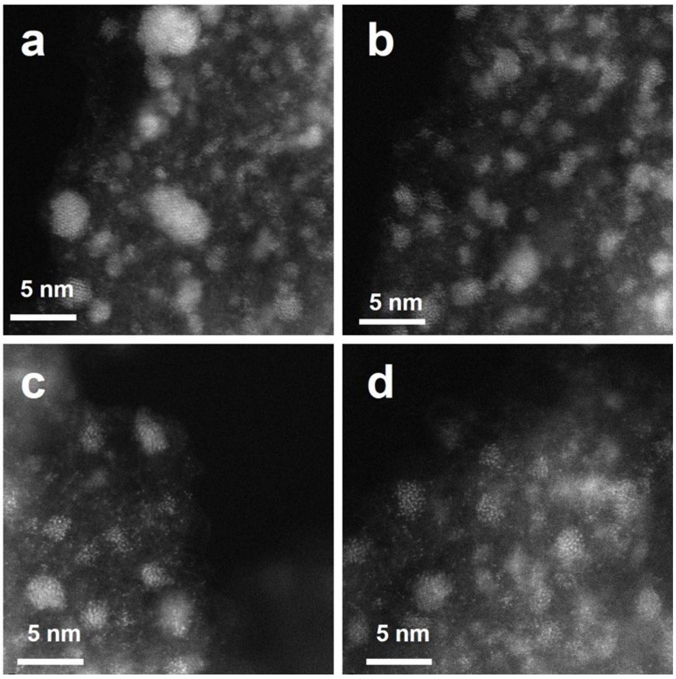

Figure S41. AC-STEM images of Catalyst Pd/C-NH_2-red 12h was synthesized via in-situ hydrogen reduction for 12 h (0.5 MPa, 60 °C).

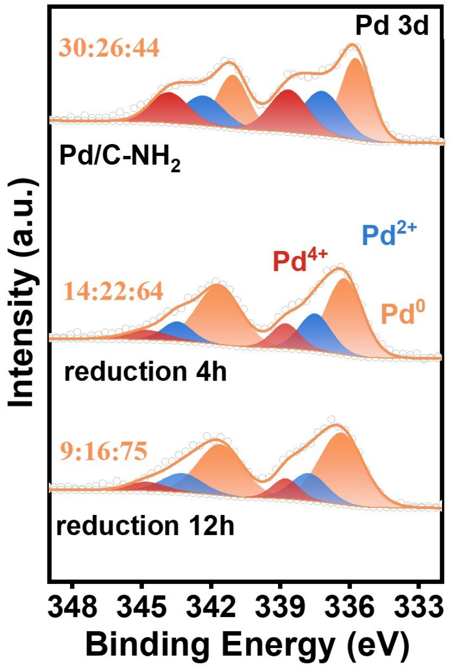

Figure S42. High-resolution Pd 3d XPS spectra of in-situ reduced catalysts.

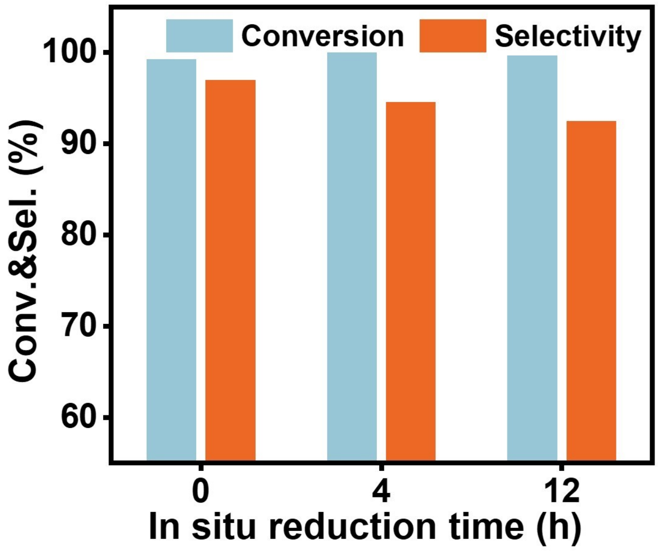

Figure S43. The performance differences in the complete conversion of in-situ reduced catalysts.

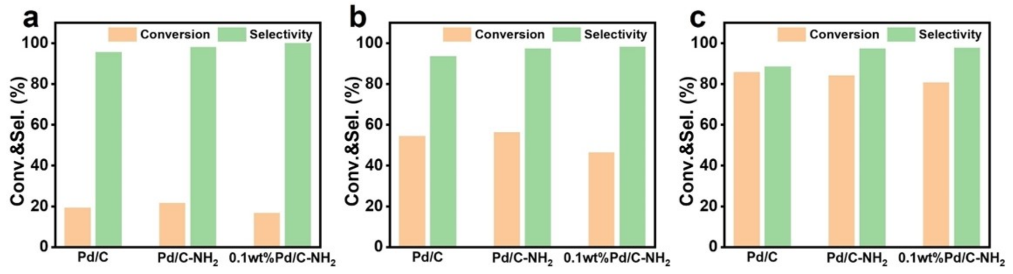

Figure S44. Comparison of selectivity at approximately the same conversion level (by varying catalyst mass or extending reaction time), adjusted to about (a) 20%, (b) 50%, and (c) 80%. The Pd/C and Pd/C-NH_2 reaction condition: 250 mg of mifepristone, 6 mg of catalyst 25 ℃, 0.1 MPa H_2. The 0.1wt.%Pd/C-NH_2 reaction condition: 250 mg of mifepristone, 60 mg of catalyst 25 ℃, 0.1 MPa H_2.

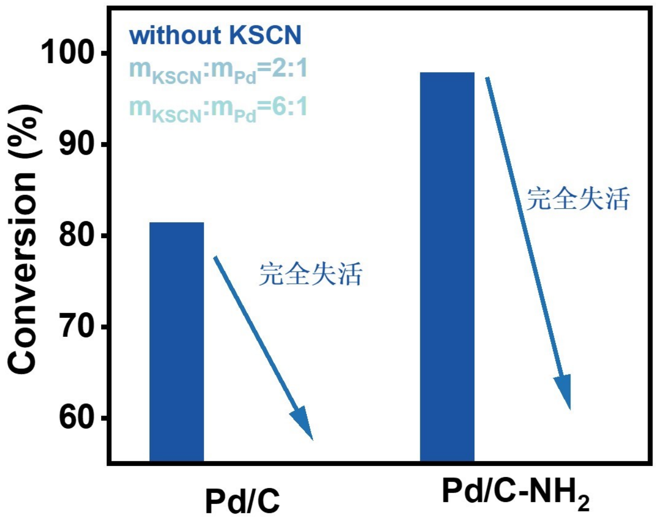

Figure S45. The poisoning effect of different n_{Pd}:n_{KSCN} ratios on catalytic performance.

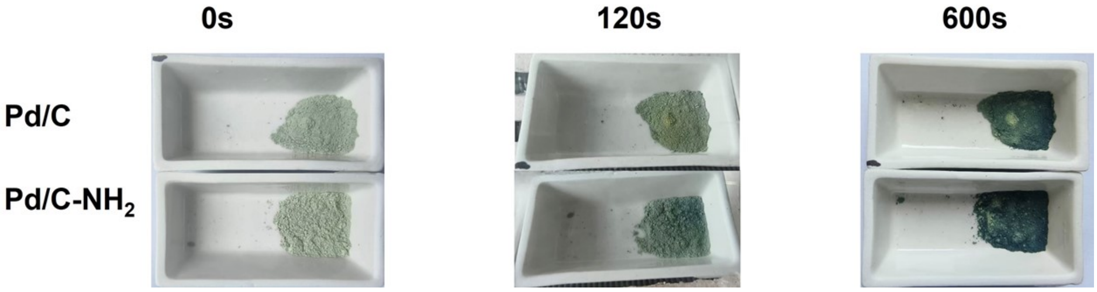

Figure S46. Hydrogen spillover discoloration experiment for Pd/C and Pd/C-NH_2 under solid-gas conditions. Reactioncondition:10 mg of catalysts, 490 mg of WO_3, 20 mL/min of pure H_2.

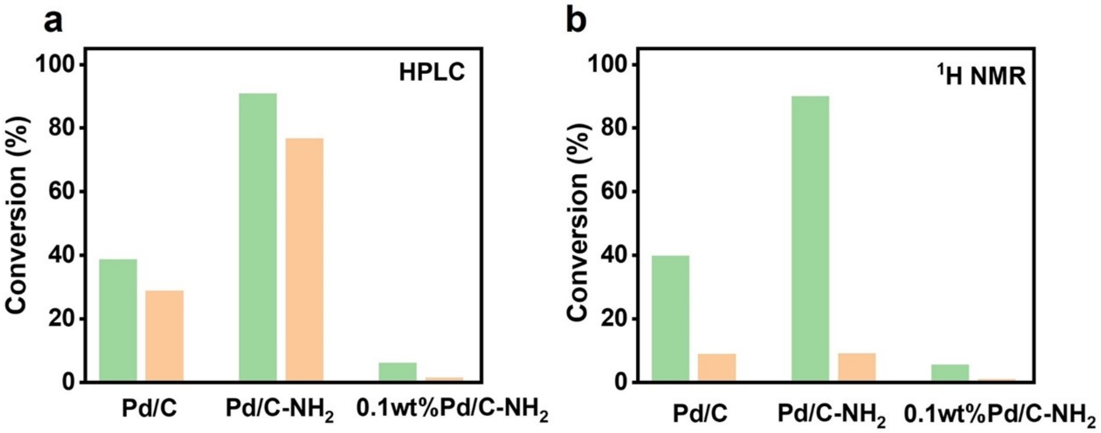

Figure S47. Catalytic performance comparison of Pd/C, Pd/C-NH_2, and 0.1 wt.% Pd/C-NH_2 under identical conditions by different analytical methods. The conversion determined by (a) high-performance liquid chromatography (HPLC), and (b) ^{1}H nuclear magnetic resonance (^{1}H NMR) spectroscopy. The green curve represents the hydrogenation reaction and yellow curve represents the deuteration reaction.

mifepristone

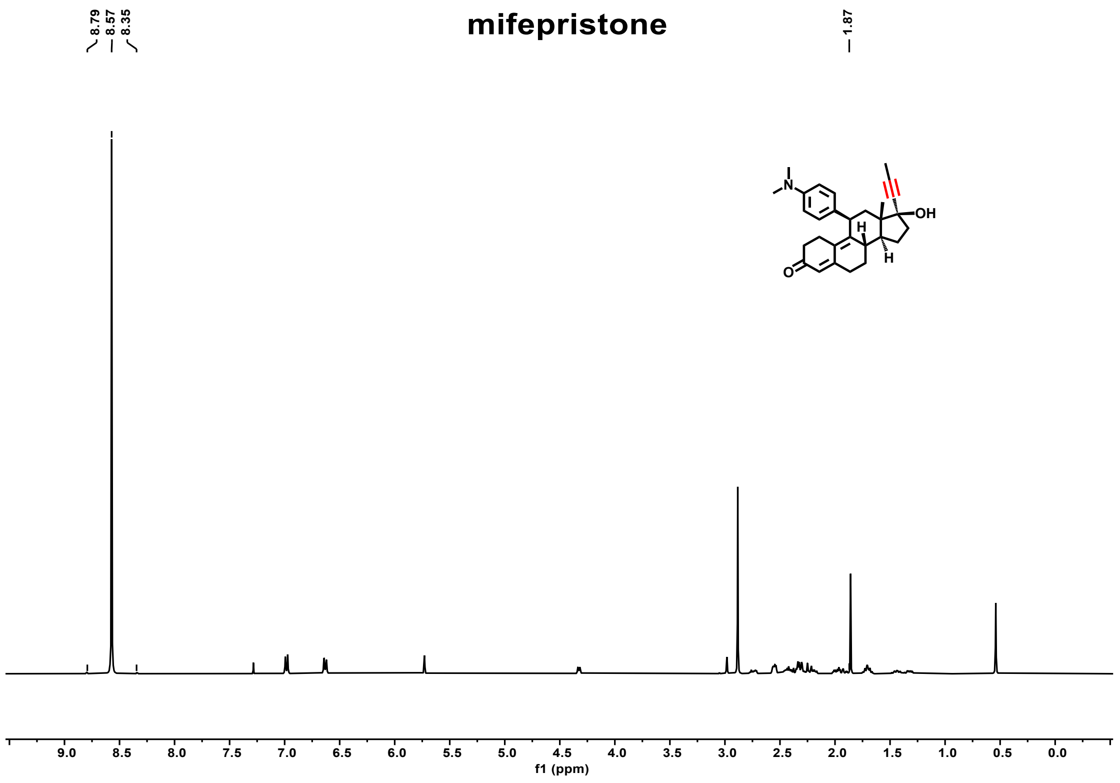

a

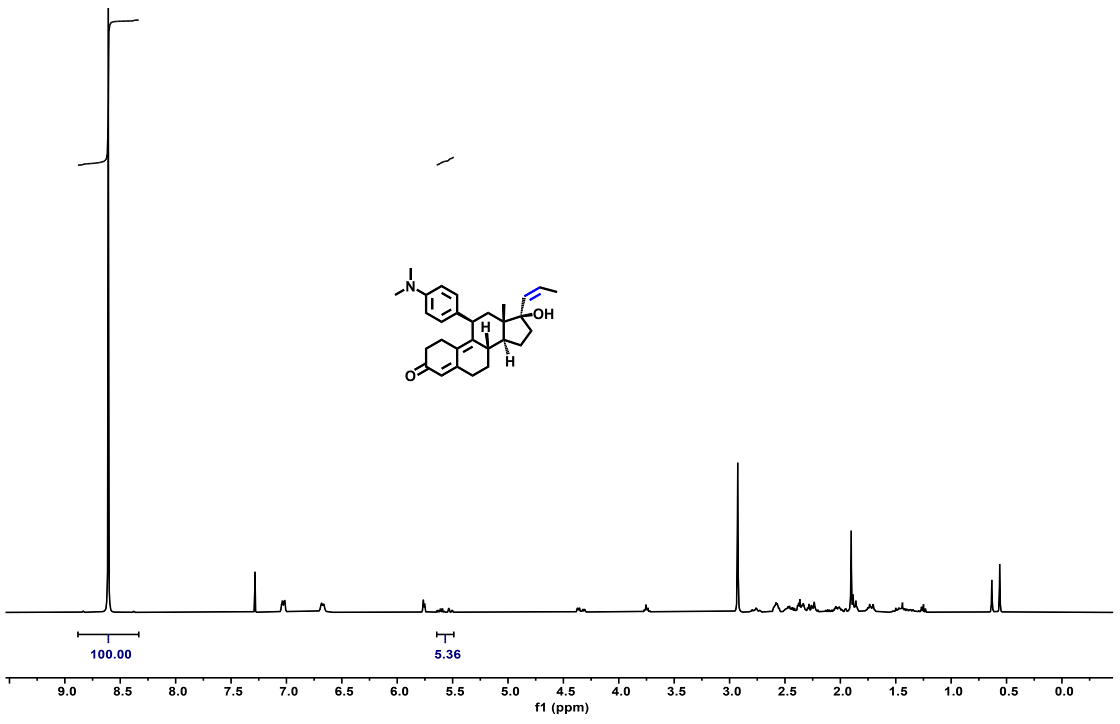

b

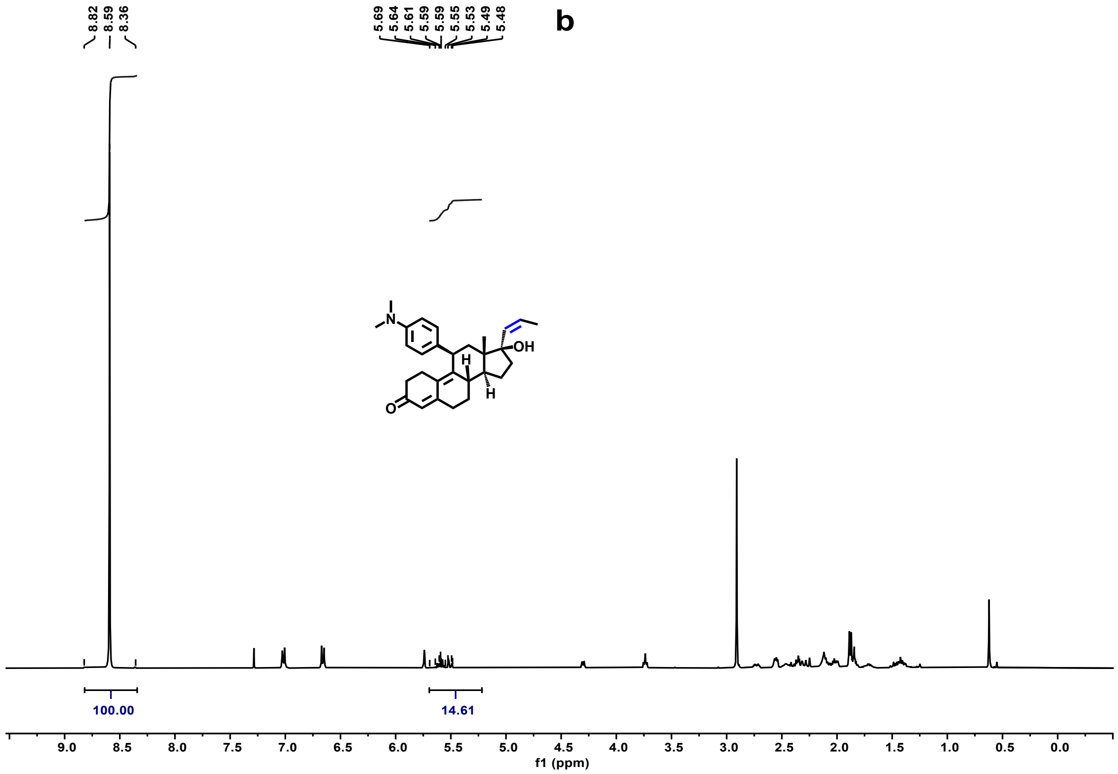

8.82
8.59
8.36
5.69
5.64
5.61
5.59
5.59
5.55
5.53
5.49
5.48
OH
H
H
N
O
100.00
14.61
9.0 8.5 8.0 7.5 7.0 6.5 6.0 5.5 5.0 4.5 4.0 3.5 3.0 2.5 2.0 1.5 1.0 0.5 0.0
f1 (ppm)

c

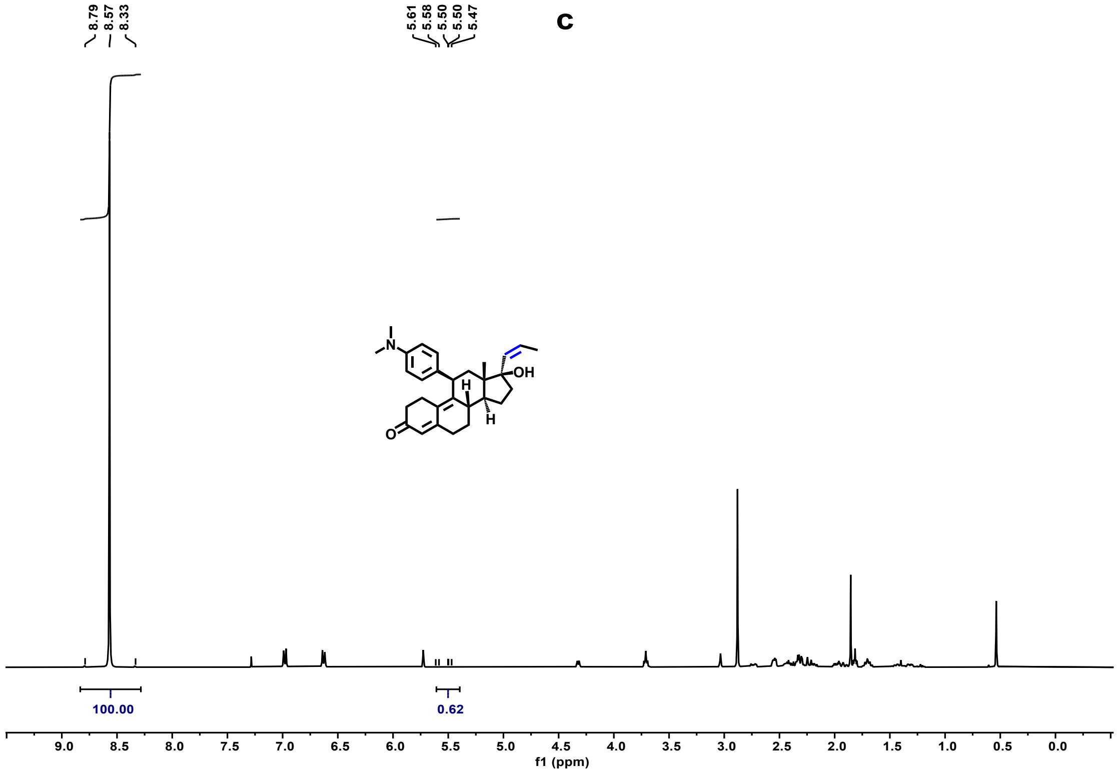

8.79
8.57
8.33
5.61
5.58
5.50
5.50
5.47
OH
H
H
N
O
100.00
0.62
9.0 8.5 8.0 7.5 7.0 6.5 6.0 5.5 5.0 4.5 4.0 3.5 3.0 2.5 2.0 1.5 1.0 0.5 0.0
f1 (ppm)

d

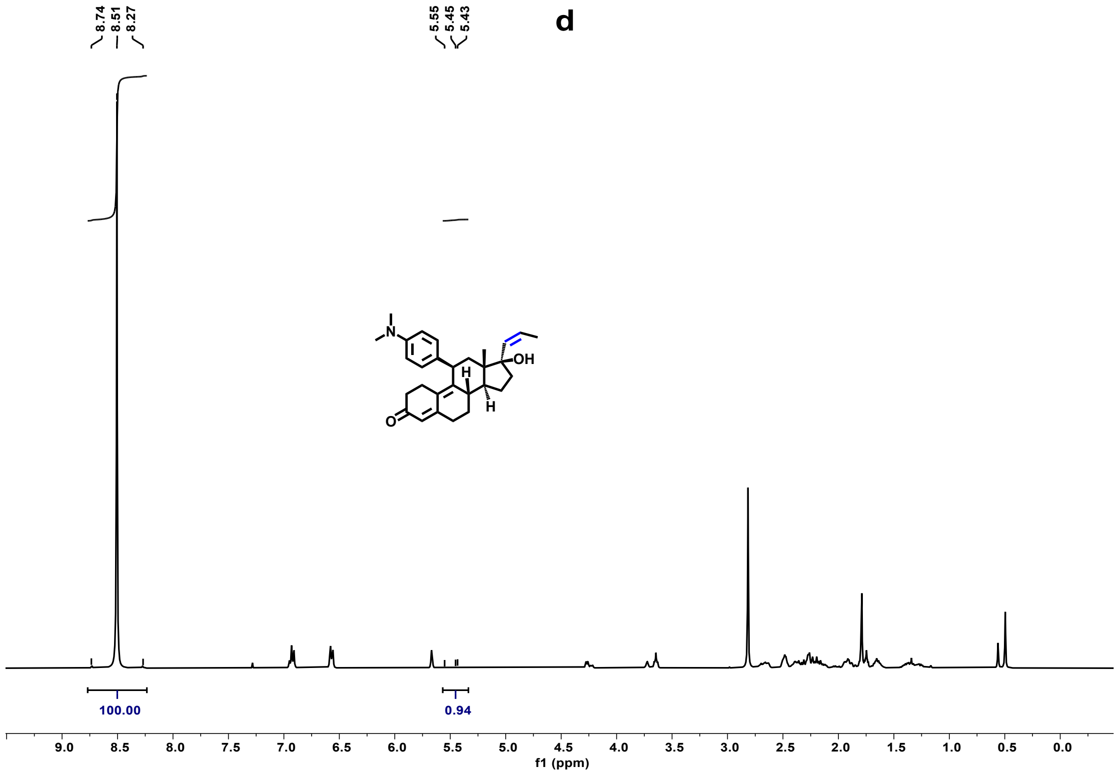
8.74
8.51
8.27
5.55
5.45
5.43
OH
H
H
H
N
O
100.00
0.94
9.0
8.5
8.0
7.5
7.0
6.5
6.0
5.5
5.0
4.5
4.0
3.5
3.0
2.5
2.0
1.5
1.0
0.5
0.0
f1 (ppm)

e

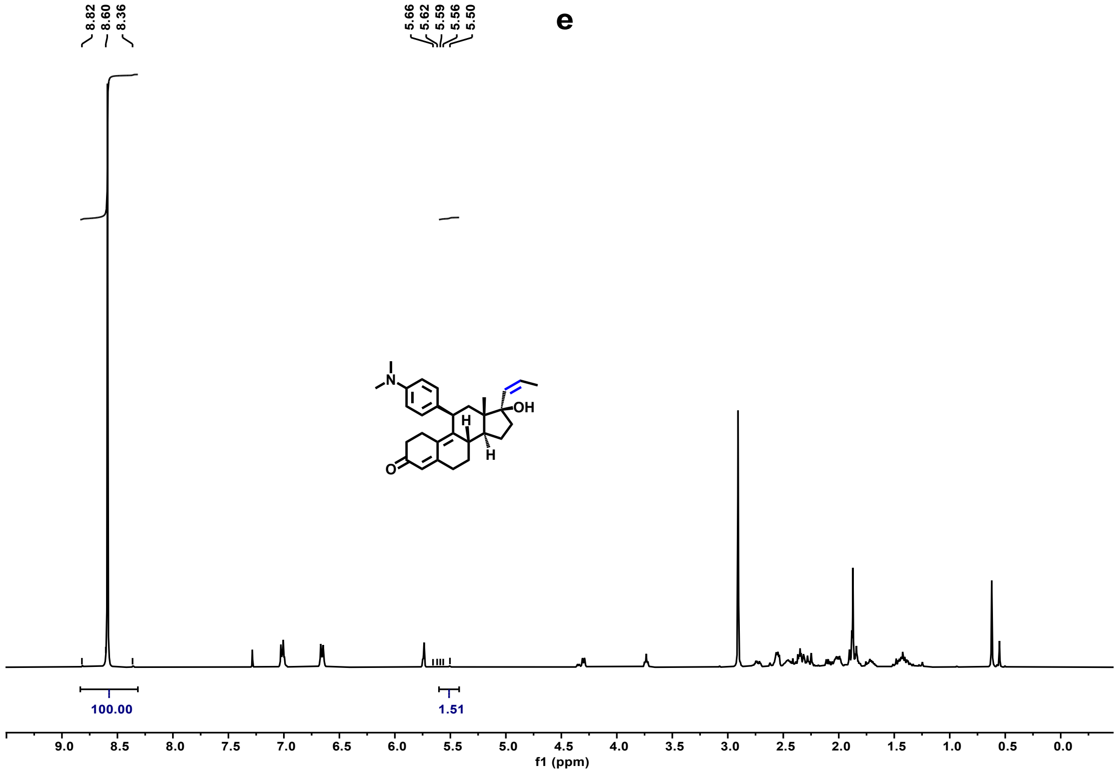
8.82
8.60
8.36
5.66
5.62
5.59
5.56
5.50
OH
H
H
H
N
O
100.00
1.51
9.0
8.5
8.0
7.5
7.0
6.5
6.0
5.5
5.0
4.5
4.0
3.5
3.0
2.5
2.0
1.5
1.0
0.5
0.0
f1 (ppm)

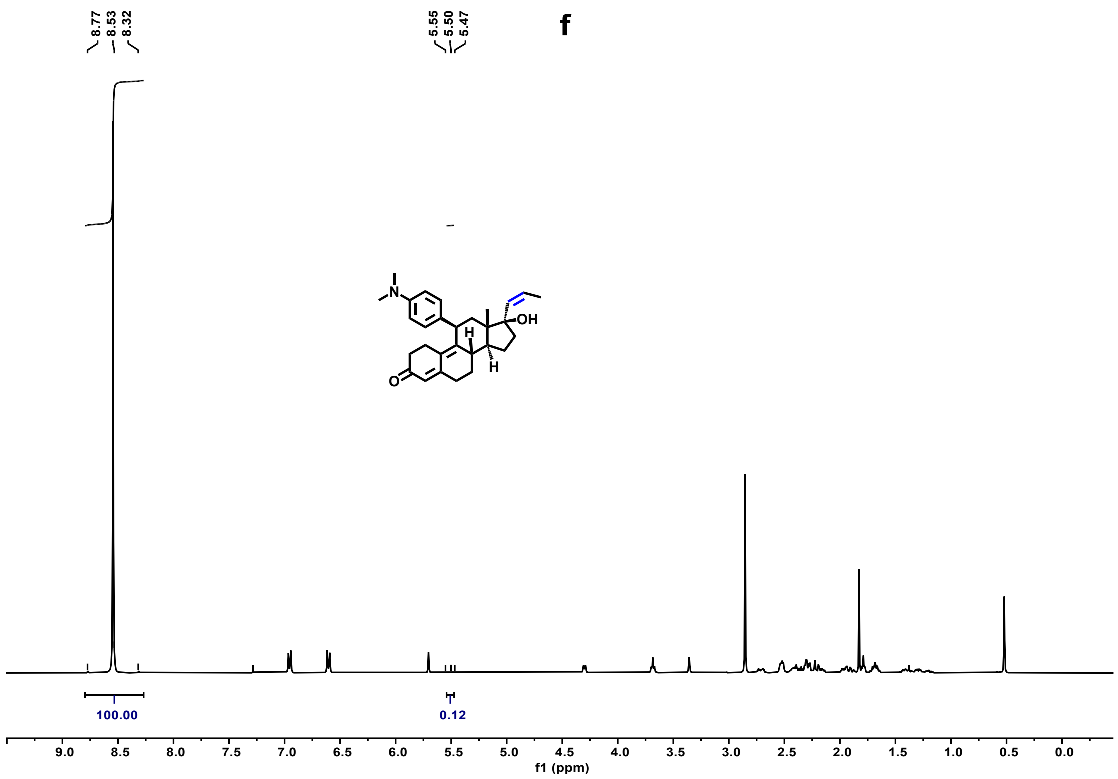

Figure S48. 1H NMR spectroscopy analyses. Mifepristone standard sample, hydrogenation of mifepristone with three catalysts: (a) Pd/C, (b) Pd/C-NH_2, (c) 0.1 wt.% Pd/C-NH_2. Deuteriumation of mifepristone with three catalysts: (d) Pd/C, (e) Pd/C-NH_2, (f) 0.1 wt.% Pd/C-NH_2. Following the general evaporation purification procedure, the corresponding product was obtained as a pale yellow solid, using deuterated chloroform as the solvent, with piperazine added as the internal standard. Reaction condition: 250 mg of mifepristone, 6 mg of catalyst, 25 °C, 0.1MPa of H_2, reaction for 10 min, 30 mg of 0.1wt.%Pd/C-NH_2, reaction for 100 min.

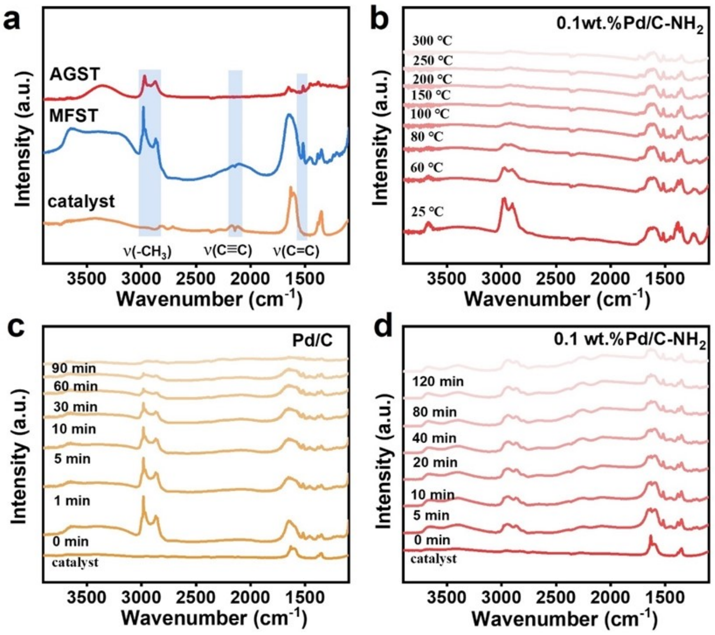

Figure S49. (a) FTIR patterns of Pd/C-NH_2 catalyst skeleton, mifepristone and anglepristone. (b) In situ FT-IR spectra showing the variation of adsorbed mifepristone on 0.1 wt.% Pd/C-NH_2 with increasing temperature. (c-d) In situ FT-IR spectra showing the variation of adsorbed mifepristone on Pd/C and 0.1 wt.% Pd/C-NH_2 with H_2 exposure time.

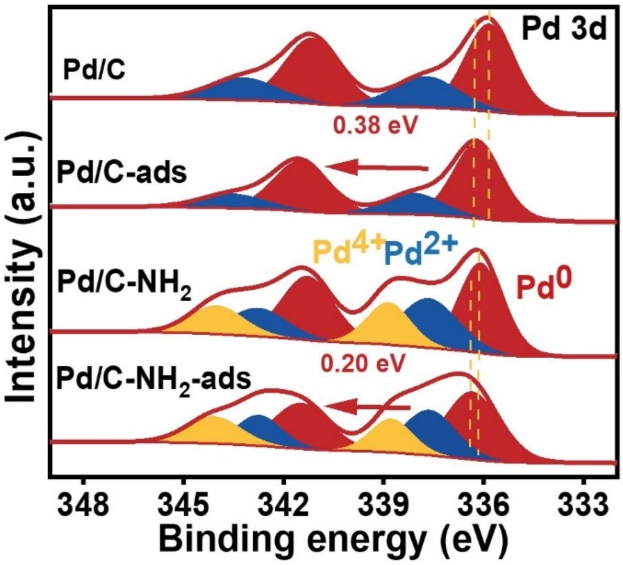

Figure S50. Parison of high-resolution Pd 3d XPS spectra of Pd/C and Pd/C-NH_2 before and after mifepristone adsorption.

Table S1. ICP-OES for different catalysts.

Catalyst	Pd loading (wt %)
Pd/C	4.95
Pd/C-NH_2	4.93
0.1 wt.%Pd/C-NH_2	0.16
Commercial Pd/C	5.00
Lindlar	5.04
Reused Pd/C	3.36
Reused Pd/C-NH_2	4.71

Table S2. Metal dispersion (determined by H_2-O_2 titration) for different catalysts.

Catalyst	H_2 uptake (μmol_{H2} g_{Pd}^{-1})	Metal dispersion (D_m, %)
Pd/C	108.36	15.55
Pd/C-NH_2	301.07	43.31
0.1 wt.%Pd/C-NH_2	21.23	94.16
Pd/C-NH_2-EDTA	111.30	48.05
Commercial Pd/C	166.20	23.59

Table S3. Peak position and intensity ratio (I_D/I_G) of D band and G band in different catalysts.

**Catalyst**	**D band (cm $^{-1}$)**	**G band (cm $^{-1}$)**	**I_D / I_G**
Pd/C	1345	1597	2.25
Pd/C-NH_2	1351	1603	2.27

Table S4. Physical properties of different catalysts.

Catalyst	S_{BET} (m^2 g^{-1}) [a]	V_{total} (cm^3 g^{-1}) [b]	Pore size (nm) [c]
C	1393.8	0.8	3.8
C-NH_2	222.1	0.3	6.9
Pd/C	986.3	0.8	5.2
Pd/C-NH_2-5h	275.5	0.3	7.0
Pd/C-NH_2	266.8	0.3	7.0
Pd/ C-NH_2-24h	146.5	0.2	7.3

[a]Specific surface areas were determined by the BET multipoint method.

[b]Total pore volumes were determined by the BJH model.

[c]Average pore sizes were determined according to the equation 4V/A.

Table S5. EXAFS data fitting results of Samples.

Sample	Path	*CN*[a]	*R*(Å)[b]	σ^2 (Å^2) [c]	ΔE_0 (eV) [d]	*R* factor
			Pd K-edge (S_0^2=0.854)			
Pd foil	Pd-Pd	12.0*	2.740±0.002	0.0057	-3.8±0.5	0.0023
PdO	Pd-O	4.1±0.2	2.020±0.007	0.0030	-3.3±1.9	0.0122
	Pd-O-Pd	2.8±0.2	3.050±0.014	0.0052	-0.5±3.1	
	Pd-O-Pd	1.8±0.3	3.457±0.024			
Pd/C-NH_2	Pd-N	3.1±0.2	2.028±0.005	0.0030	-3.9±1.0	0.0075
	Pd-Pd	3.0±0.1	2.739±0.004	0.0063		

[a]*CN*, coordination number.

[b]*R*, the distance between absorber and backscatter atoms.

[c]σ^2, the Debye Waller factor value.

[d]ΔE_0, inner potential correction to account for the difference in the inner potential between the sample and the reference compound.

R factor indicates the goodness of the fit. $S0^2$ was fixed to 0.854, according to the experimental EXAFS fit of Pd foil by fixing *CN* as the known crystallographic value. * This value was fixed during EXAFS fitting, based on the known structure of Pd. Fitting conditions: *k* range：3.0 - 13.0; *R* range: 1.3-3.0; fitting space: R space; *k*-weight = 3. A reasonable range of EXAFS fitting parameters: $0.700 < S_0^2 < 1.000$; $CN > 0$; $\sigma^2 > 0$ Å^2; $|\Delta E_0| < 15$ eV; *R* factor < 0.02.

Table S6. Comparison of TOF values of different catalysts.

Reaction condition: 250 mg of mifepristone, 6 mg of catalyst, 25 °C, 0.1 MPa H_2.

Catalyst	Mass (mg)	t/min	Conversion (%)	Selectivity (%)	TOF/h^{-1}
Pd/C	6	5	19	96	3094
Pd/C-NH_2	6	1	13	98	3675
0.1wt%Pd/C-NH_2	30	360	8	100	18
Commercial Pd/C	6	3	15	99	2695
Lindlar	6	30	10	99	205

Table S7. Comparison of phenylacetylene hydrogenation activity of different catalysts. Pd/C-NH_2 catalyst reaction condition: 250 mg of mifepristone, 6 mg of catalyst, 25 °C, 0.1 MPa H_2.

Catalyst	T/°C	P_{H2}/MPa	Solvent	Conv.	Sel.	TOF/h^{-1}	Stability	Additive	Reference
Pd/C-NH_2	25	0.1	THF+H_2O	99%	97%	3675	10	no	This work
PdCx@S-1	25	0.2	EtOH	99%	99%	513	5	no	1
Pd-Pb NSs	80	0.8	EtOH	100%	96%	2256	6	no	2
$Pd_{4.5}Se$ NC	110	0.1	DMF	100%	98%	351.4	10	no	3
Pd-Ag (3:1)-PAM/ZnO	40	0.1	EtOH	91%	88%	2448	10	no	4
Pd@PPy-600	35	atm	EtOH	99%	96%	4912	5	no	5
PdCo-QT	25	0.1	EtOH	96%	97%	19.1	5	no	6
Pd SAC-Au	25	0.1	EtOH	99.99%	90%	3964	6	no	7
PdNi/SiO_2	30	atm	toluene	100%	100%	4002	5	no	8
PdO/C_{PYMO}	40	$NaBH_4$	H_2O	100%	92%	800	-	no	9
Pd-Ru@ZIF-8	100	0.1	EtOH	98%	96%	2188	5	no	10
Pd_1Fe SAA@PC	25	0.1	CH_3OH	99.9%	98.9%	14310	10	no	11
Pd/AZ−B	25	0.2	EtOH	99%	95%	24198	5	no	12
Pd_1/Ni@G	30	0.2	EtOH	100%	93%	7074	5	no	13
Pd_1@ZSM-5	65	1	Normal hexane	100%	96%	33582	10	no	14
Pd-Mn/NC	60	1.5	Normal hexane	99%	95%	13080	5	no	15
Pd@TMC1	25	atm	EtOH	95%	95%	6334	10	no	16
fcc Ag@hcp Ni	70	0.3	DMF	100%	95%	8241.8	-	no	17
Pt-MXene	50	0.3	EtOH+N-decane	50.6%	82.4%	10548	-	no	18
Pd/$BaTiO_{(3-x)}H_x$	25	0.4	CH_3OH	99.7%	99.7%	9180	8	no	19
a-CuPd	20	0.1	EtOH	99.1%	96.2%	6004	5	no	20

Table S8. Valence state distribution and TOF values of different catalysts. Reaction condition: 250 mg mifepristone, 6 mg of catalyst, 25 °C, 0.1 MPa H_2.

Catalyst	Pd^0	Pd^{2+}	Pd^{4+}	Time/min	Conversion	Selectivity	TOF/h^{-1}
Pd/C	61	39	/	5	19%	96%	3094
Pd/C-NH_2	44	26	30	1	13%	98%	3675
EtOH : H_2O=45:5	51	41	8	5	8%	97%	478
EtOH :H_2O=30:20	50	34	16	2	8%	97%	1189
EtOH :H_2O=20:30	45	36	19	2	11%	97%	1521
400 ℃	48	32	20	3	16%	98%	1522
800 ℃	41	35	14	2	9%	98%	1371
1000 ℃	54	37	9	6	11%	98%	534
Pd/C-NH_2-red 4h	49	33	18	2	10%	98%	1492
Pd/C-NH_2-red 12h	50	43	7	10	11%	98%	315

Table S9. The conversion detected by HPLC and ^{1}H NMR when H_2/D_2 is introduced under the same conditions. Reaction condition: 250 mg of mifepristone, 6 mg of catalyst, 25 ℃, 0.1MPa of H_2, reaction for 10 min, 30 mg of 0.1wt.%Pd/C-NH_2, reaction for 100 min.

Catalyst	Gas	HPLC Conversion (%)	NMR Conversion (%)
Pd/C	H_2	39	40
Pd/C	D_2	29	9
Pd/C-NH_2	H_2	91	90
Pd/C-NH_2	D_2	77	9
0.1wt.%Pd/C-NH_2	H_2	6	6
0.1wt.%Pd/C-NH_2	D_2	2	1

References

1. Bai, R.; He, G.; Li, L.; Zhang, T.; Li, J.; Wang, X.; Wang, X.; Zou, Y.; Mei, D.; Corma, A.; Yu, J. Encapsulation of Palladium Carbide Subnanometric Species in Zeolite Boosts Highly Selective Semihydrogenation of Alkynes. Angew. Chem. Int. Ed. 2023, *62* (48), e202313101.

2. Shen, C.; Ji, Y.; Wang, P.; Bai, S.; Wang, M.; Li, Y.; Huang, X.; Shao, Q. Interface Confinement in Metal Nanosheet for High-Efficiency Semi-Hydrogenation of Alkynes. *ACS Catal.* 2021, *11* (9), 5231-5239.

3. Wang, M.; Liang, L.; Liu, X.; Sun, Q.; Guo, M.; Bai, S.; Xu, Y. Selective semi-hydrogenation of alkynes on palladium-selenium nanocrystals. *J. Catal.* 2023, *418*, 247-255.

4. Zharmagambetova, A.; Auyezkhanova, A.; Talgatov, E.; Jumekeyeva, A.; Buharbayeva, F.; Akhmetova, S.; Myltykbayeva, Z.; Nieto, J. M. L. Synthesis of polymer protected Pd–Ag/ZnO catalysts for phenylacetylene hydrogenation. *J. Nanopart. Res.* 2022, *24* (12).

5. Zhang, R.; Liu, Z.; Zheng, S.; Wang, L.; Zhang, L.; Qiao, Z.-A. Pyridinic Nitrogen Sites Dominated Coordinative Engineering of Subnanometric Pd Clusters for Efficient Alkynes' Semihydrogenation. *Adv. Mater.* 2023, *35* (11), 2209635.

6. Martinez, J. S.; Carpisassi, L.; Egea, G.; Mazarío, J.; Lopes, C. W.; Mora-Moreno, C.; Trasobares, S.; Vaccaro, L.; Calvino, J. J.; Agostini, G.; Oña-Burgos, P. MOF-Derived PdCo and PdMn Systems as Versatile Catalysts in Alkyne Semihydrogenation. *ACS Catal.* 2025, *15* (9), 7263-7282.

7. Zhu, A.; Zhao, N.; Mao, Y.; Yang, L.; Qi, J.; Alomar, T. S.; AlMasoud, N.; Xie, W. Nonequilibrium carriers trigger hydrogen spillover for the highly efficient semihydrogenation of alkynes under ambient conditions. *eScience* 2025, 100481.

8. Zhang, Y.; Xu, C.; Wu, Y.; Li, S. Enhanced Catalytic Activity of PdNi Dilute Nanoalloy for Selective Phenylacetylene Hydrogenation. *J. Nanoelectron. Optoelectron.* 2021, *16* (3), 380-386.

9. Augustyniak, A. W.; Trzeciak, A. M. Pd-Nanocomposites Formed by Calcination of [Pd(2-pymo)2]n Framework as Catalysts of Phenylacetylene Semihydrogenation in Water. ChemCatChem 2021, *13* (9), 2145-2151.

10. Li, Z.; Hu, M.; Liu, J.; Wang, W.; Li, Y.; Fan, W.; Gong, Y.; Yao, J.; Wang, P.; He, M.; Li, Y.

Mesoporous silica stabilized MOF nanoreactor for highly selective semi-hydrogenation of phenylacetylene via synergistic effect of Pd and Ru single site. *Nano Res.* 2021, *15* (3), 1983-1992.

11. Sun, Z.; Li, C.; Lin, J.; Guo, T.; Song, S.; Hu, Y.; Zhang, Z.; Yan, W.; Wang, Y.; Wei, Z.; Zhang, F.; Zheng, K.; Wang, D.; Li, Z.; Wang, S.; Chen, W. Lattice Strain and Mott-Schottky Effect of the Charge-Asymmetry Pd_1Fe Single-Atom Alloy Catalyst for Semi-Hydrogenation of Alkynes with High Efficiency. *ACS Nano* 2024, 18 (20), 13286-13297.

12. Bai, R.; He, G.; Li, J.; Li, L.; Zhang, T.; Wang, X.; Zhang, W.; Zou, Y.; Zhang, J.; Mei, D.; Corma, A.; Yu, J. Heteroatoms-Stabilized Single Palladium Atoms on Amorphous Zeolites: Breaking the Tradeoff between Catalytic Activity and Selectivity for Alkyne Semihydrogenation. *Angew. Chem. Int. Ed.* 2024, 63 (44), e202410017.

13. Zhao, L.; Qin, X.; Zhang, X.; Cai, X.; Huang, F.; Jia, Z.; Diao, J.; Xiao, D.; Jiang, Z.; Lu, R.; Wang, N.; Liu, H.; Ma, D. A Magnetically Separable Pd Single-Atom Catalyst for Efficient Selective Hydrogenation of Phenylacetylene. *Advanced Materials* 2022, 34 (20).

14. Liu, H.; Li, J.; Liang, X.; Ren, H.; Yin, H.; Wang, L.; Yang, D.; Wang, D.; Li, Y. Encapsulation of Pd Single-Atom Sites in Zeolite for Highly Efficient Semihydrogenation of Alkynes. *J Am Chem Soc* 2024, 146 (34), 24033-24041.

15. Liu, H.; Zhu, P.; Yang, D.; Zhong, C.; Li, J.; Liang, X.; Wang, L.; Yin, H.; Wang, D.; Li, Y., Pd-Mn/NC Dual Single-Atomic Sites with Hollow Mesopores for the Highly Efficient Semihydrogenation of Phenylacetylene. *J Am Chem Soc* 2024, 146 (3), 2132-2140.

16. Zhang, M.; Liu, R.; He, Z.; Wang, L.; Zhang, X.; Li, G. Large Triazine Organic Cage Encapsulating Pd Nanoclusters for Selective Hydrogenation of Alkynes. *ACS Catalysis* 2025, 15 (14), 12247-12259.

17. Su, J.; Ji, Y.; Geng, S.; Li, L.; Liu, D.; Yu, H.; Song, B.; Li, Y.; Pao, C. W.; Hu, Z.; Huang, X.; Lu, J.; Shao, Q. Core-Shell Design of Metastable Phase Catalyst Enables Highly-Performance Selective Hydrogenation. *Adv Mater* 2024, 36 (7), e2308839.

18. Little, J. M.; Chen, A.; Kamali, A.; Akash, T. S.; Park, C. S.; Liu, D.; Das, S.; Woehl, T. J.; Chen, P. Y. Drying Controlled Synthesis of Catalytic Metal Nanocrystals Within 2D-Material Nanoconfinements. *Advanced Functional Materials* 2024, 35 (6).

19. Miyazaki, M.; Ogasawara, K.; Nakao, T.; Sasase, M.; Kitano, M.; Hosono, H. Hexagonal

$BaTiO_{(3-x)}H_x$ Oxyhydride as a Water-Durable Catalyst Support for Chemoselective Hydrogenation. *J Am Chem Soc* 2022, 144 (14), 6453-6464.

20. Yang, H.; Yan, B.; Xue, Y.; Guo, T.; Wang, Z.; Teo, G.; Hu, P.; Liu, L.; Guo, L. Amorphous monolayer CuPd catalysts for
selective semihydrogenation. *Sci. Adv.* 2025, 11 (34), eadx8081.
